# Executive functioning and reading achievement in school: a study of Brazilian children assessed by their teachers as “poor readers”

**DOI:** 10.3389/fpsyg.2014.00550

**Published:** 2014-06-10

**Authors:** Pascale M. J. Engel de Abreu, Neander Abreu, Carolina C. Nikaedo, Marina L. Puglisi, Carlos J. Tourinho, Mônica C. Miranda, Debora M. Befi-Lopes, Orlando F. A. Bueno, Romain Martin

**Affiliations:** ^1^ECCS Research Unit, University of LuxembourgWalferdange, Luxembourg; ^2^Instituto de Psicologia, Universidade Federal da BahiaSalvador, Brazil; ^3^Departamento de Psicobiologia, Universidade Federal de São PauloSão Paulo, Brazil; ^4^Departamento de Fisioterapia Fonoaudiologia e Terapia Ocupacional, Universidade de São PauloSão Paulo, Brazil

**Keywords:** executive function, reading, working memory, cognitive flexibility, selective attention, inhibition, poverty, learning difficulties

## Abstract

This study examined executive functioning and reading achievement in 106 6- to 8-year-old Brazilian children from a range of social backgrounds of whom approximately half lived below the poverty line. A particular focus was to explore the executive function profile of children whose classroom reading performance was judged below standard by their teachers and who were matched to controls on chronological age, sex, school type (private or public), domicile (Salvador/BA or São Paulo/SP) and socioeconomic status. Children completed a battery of 12 executive function tasks that were conceptual tapping cognitive flexibility, working memory, inhibition and selective attention. Each executive function domain was assessed by several tasks. Principal component analysis extracted four factors that were labeled “Working Memory/Cognitive Flexibility,” “Interference Suppression,” “Selective Attention,” and “Response Inhibition.” Individual differences in executive functioning components made differential contributions to early reading achievement. The Working Memory/Cognitive Flexibility factor emerged as the best predictor of reading. Group comparisons on computed factor scores showed that struggling readers displayed limitations in Working Memory/Cognitive Flexibility, but not in other executive function components, compared to more skilled readers. These results validate the account that working memory capacity provides a crucial building block for the development of early literacy skills and extends it to a population of early readers of Portuguese from Brazil. The study suggests that deficits in working memory/cognitive flexibility might represent one contributing factor to reading difficulties in early readers. This might have important implications for how educators might intervene with children at risk of academic under achievement.

## Introduction

Reading is a complex cognitive task that depends on a range of component skills. It is now well established that children's phonological awareness, letter-sound knowledge and broader oral language abilities play an important role in their reading development (Carroll et al., 2003; Muter et al., 2004; Nation et al., 2004; Rose, 2006; Nation et al., 2010; Fricke et al., 2013). More recently, executive functioning skills have been put forward as another crucial building block for literacy development. Children who struggle to read fluently or do not understand well what they read often have problems with their executive functions (Reiter et al., 2005; Sesma et al., 2009; Locascio et al., 2010; Pimperton and Nation, 2010, 2014). Much debate remains, however, regarding the exact nature and degree of executive functioning difficulties experienced by struggling readers.

The term “executive function” encompasses a collection of cognitive processes that people use to coordinate and control their thoughts and actions, particularly in novel situations, and that are crucial for higher-order problem solving and goal-directed behavior (Zelazo et al., 2008; Zelazo and Carlson, 2012). Executive functioning is often assessed by “complex tasks” such as the Tower of London or the Wisconsin Card Sorting Test that involve several lower-level executive functioning abilities. Studies using such complex executive function tasks generally report correlations with literacy (Hooper et al., 2002; Sesma et al., 2009). Recent theoretical models posit that in adults, different executive functions constitute distinct, yet related, components (Miyake et al., 2000; Friedman et al., 2008). There is also some evidence suggesting that executive functions represent a set of dissociable abilities in children, although the nature of these factors differs widely across studies and developmental populations (Lehto et al., 2003; Senn et al., 2004; Huizinga et al., 2006; St. Clair-Thompson and Gathercole, 2006; Van der Sluis et al., 2007; Wiebe et al., 2008; Rose et al., 2011; Steele et al., 2012). Cognitive flexibility, working memory and inhibitory control are regarded by many as core components of executive functioning because they are relatively well defined conceptually, often emerge as dissociable constructs in factor-analytic models and have been shown to be implicated in performance on more complex executive function tasks (Baddeley, 1996; Roberts and Pennington, 1996; Rabbitt, 1997; Miyake et al., 2000; Lehto et al., 2003).

A concept closely related to executive function is attention and many descriptions of executive functioning also include subfunctions of attention (Klenberg et al., 2001; Manly et al., 2001; Breckenridge et al., 2013; Loher and Roebers, 2013). In an attempt to separate different executive function components through exploratory factor analyses in 7- to 12-year-old children, Klenberg et al. (2001) reported that inhibition, auditory attention, selective visual attention and fluency clustered into separate factors. Another exploratory factor analysis involving 11-year-olds identified a two factor structure: one associated with working memory and one with inhibition. The study also included measures of cognitive flexibility that failed, however, to relate to a third distinct executive factor (St. Clair-Thompson and Gathercole, 2006).

Working memory has been described as a cognitive system of multiple components that is used to store and work with information in mind for brief periods of time (Baddeley, 2000). Most theorists agree that working memory comprises mechanisms devoted to the maintenance of information over a short period of time, also referred to as short-term memory and processes responsible for executive control that regulate and coordinate those maintenance operations (Engle et al., 1999). Whereas so-called simple span tasks mainly tap into the short-term storage component of the working memory system, performance on complex span tasks, that involve the simultaneous processing and storage of information, has been argued to rely on both central executive resources and domain-specific short-term storage systems (Duff and Logie, 2001). Some studies show a large or even complete overlap between simple and complex span tasks of working memory (Alloway et al., 2006), and it has been claimed by some that both types of measures essentially tap into the same underlying cognitive process (Unsworth and Engle, 2007). There is some evidence for discrete verbal and visuo-spatial working memory components (Shah and Miyake, 1996; Friedman and Miyake, 2000; Jarvis and Gathercole, 2003; Kane et al., 2004). In children it has been shown that verbal and visuo-spatial working memory tasks can relate to the same underlying factor while at the same time accounting for unique variance in academic achievement (St. Clair-Thompson and Gathercole, 2006). Verbal and visuo-spatial working memory measures might thus reflect partly domain general mechanisms and partly the contribution of modality specific storage systems (Baddeley and Logie, 1999; St. Clair-Thompson and Gathercole, 2006).

Cognitive flexibility (also known as task switching or set shifting) refers to the ability to flexibly adapt thoughts or actions as demanded by the situation (Cragg and Nation, 2008). It is generally assessed by tasks that consist of different conditions and that require subjects to switch from one condition to another in response to an external cue. Inhibitory control denotes processes which are involved in suppressing dominant but irrelevant stimuli or responses (Nigg, 2000). Several subtypes of inhibition have been proposed (Barkley, 1997; Friedman and Miyake, 2004; Martin-Rhee and Bialystok, 2008; Nigg, 2000). For example, Martin-Rhee and Bialystok (2008) distinguished tasks of response inhibition that require to override habitual responses to univalent displays (e.g., Go/No-Go paradigm) from tasks of interference suppression that are based on bivalent displays in which two presented features indicate potentially conflicting responses (e.g., Flanker paradigm). Selective attention refers to the ability to focus on particular information and to screen out irrelevant stimuli. It is often assessed through visual search paradigms in which target objects or features must be located among other distracters (Manly et al., 2001; Scerif et al., 2004).

Emerging research supports the idea of the contribution made by executive functioning to reading development. Working memory has been the most frequently studied and numerous findings point toward a positive relationship between performance in working memory tasks and reading proficiency (Gathercole et al., 2006a,b; St. Clair-Thompson and Gathercole, 2006; Swanson and Sachse-Lee, 2001; Swanson et al., 2004, 2011; Welsh et al., 2010). Whereas verbal short-term memory tasks have been linked consistently to decoding skills, complex span tasks have been found to make significant contributions to reading comprehension (Swanson and Berninger, 1995; Engel de Abreu and Gathercole, 2012). Working memory has also been linked to other areas of academic learning. Children with low working memory capacity often make poor general academic progress, leading to the hypothesis that working memory might act as a bottleneck for learning (Gathercole and Alloway, 2008). Cognitive flexibility has also been associated with reading ability (Hooper et al., 2002; Van der Sluis et al., 2007; Welsh et al., 2010; Cartwright, 2012). In a study from the US, Welsh et al. (2010) found that preschoolers' cognitive flexibility skills predicted their decoding and word recognition abilities at the end of kindergarten. Similarly, Van der Sluis et al. (2007) have shown that cognitive flexibility was positively linked to word-reading efficiency in 9–12-year-old children from the Netherlands.

Few studies have investigated inhibition and selective attention in relation to reading. Inhibitory processes have been implicated in reading in some studies (De Beni et al., 1998; De Beni and Palladino, 2000) but not in others (Lan et al., 2011). Lan et al. (2011) explored inhibition, working memory and selective attention cross-culturally in preschool children from China and the US and found that selective attention was the most robust predictor for letter–word identification in both countries. In contrast, in a longitudinal study on 3–6-year-olds from the UK, Steele et al. (2012) did not find a relationship between the ability to select and sustain attention and word recognition a year later. There is some evidence that struggling readers display limitations in tasks of selective attention (Sireteanu et al., 2008; Romani et al., 2011). For example Casco et al. (1998) showed that 11–12-year-old children with the lowest performance on a selective attention task achieved significantly lower scores in reading fluency than children with the highest selective attention abilities.

Other research exploring the contribution of executive functioning to reading has focused on clinical groups. Reiter et al. (2005) found that compared to their typically developing peers, children with dyslexia manifested deficits on measures of verbal and visuo-spatial working memory, inhibition, planning, and cognitive flexibility. An increasing body of research also suggests that specific reading comprehension difficulties are linked to executive dysfunction (Nation et al., 1999; Cain, 2006; Sesma et al., 2009; Borella et al., 2010; Locascio et al., 2010; Pimperton and Nation, 2010). In a study from the US, Locascio et al. (2010) found that children with specific reading comprehension difficulties (“poor comprehenders”) were impaired on tasks tapping planning and visuo-spatial working memory. Findings from the UK indicate, however, that poor comprehenders show domain specific working memory and inhibitory deficits that are restricted to the verbal domain (Pimperton and Nation, 2010).

In sum, differences in executive functioning have been reported in good and poor readers but it remains unclear which specific executive function components might be affected. Few studies have included a comprehensive battery of tasks tapping into various facets of executive functioning ability. Furthermore, previous studies have focused almost exclusively on English speaking children from the US or the UK. Little is known about the relationship between specific components of executive functioning and reading in other cultural and linguistic contexts.

### The present study

This study was conducted in Brazil with typically developing children in the early primary school years. Children in Brazil learn to read and write in Portuguese, a Romance language that is spoken by approximately 200 million people world-wide. The Portuguese orthographic code is more transparent than the English one, although less transparent than other European languages such as German or Italian (Pinheiro, 1995). Despite major improvement over the last decade, many students in Brazil perform below expected levels of literacy. The latest figures from the OECD “Programme for International Student Assessment” (PISA) indicate that Brazil ranks 55 out of 65 countries on reading, with half of the country's students performing below the basic proficiency level (OECD, 2013). Constructivist teaching methods (also known as the “whole language” approach) represent the dominant approach to literacy instruction in Brazil (Abadzi, 2006; Belintane, 2006). This approach is based on the belief that children discover the alphabetic code spontaneously in the course of reading and writing, and stands in contrast to the skill-based phonics approach that is used widely in English-speaking countries (National Institute of Child Health and Human Development, 2000; Ehri et al., 2001).

Our study explored working memory, cognitive flexibility, inhibition and selective attention in a large sample of young children from a range of social backgrounds. Each of these executive function domains was assessed with multiple measures that were carefully selected from the cognitive neuroscience literature and that are widely used in research and clinical settings to measure processes related to executive functioning in children. The objective was to choose relatively simple tasks that conceptually tap into isolated executive function components. The first step toward understanding the nature of the contribution made by different components of executive functioning to reading is to explore whether these theoretically distinguishable executive functions are actually discernible as distinct factors in a population of Brazilian children from a range of demographic backgrounds. Notably, our sample was ethnically and socioeconomically diverse and approximately 50% of the children lived below the poverty line.

A major interest was to explore the executive function profile of children who were assessed by their teachers as low reading achievers but without a diagnosed learning disability. There is without a doubt much controversy over whether teachers can identify reliably those children with reading problems. In an educational system such as that in Brazil, teachers' judgment of children's level of achievement is, however, crucial because grade (i.e., school year) repetition is common practice and is primarily initiated by the school on the basis of teachers' judgment of children's levels of attainment (Bruns et al., 2011). Low achieving students are held in the same grade for an extra year rather than being promoted to a higher grade along with their age peers. In Brazil almost 25% of students in the first grade repeat a year (PREAL, 2009) with children from the poorest segments of society being most affected (Bruns et al., 2011). Costs associated with grade repetition in Brazil are among the highest in the world (OECD, 2011). According to a recent World Bank estimate, Brazil spends approximately 12% of its total basic education expenditure on grade repetition (Bruns et al., 2011). The problem of grade repetition is however not restricted to Brazil; approximately 32.2 million children in primary education worldwide repeat a grade (UNESCO, 2012). A major reason for grade repetition around the world is low levels of academic performance.

There is a general consensus that the ability to read is a fundamental educational skill that forms the basis for all future learning. Children need to be able to read well in order to engage fully in the classroom and learn. Today many students across the developing world have reading difficulties that can have tremendous long-term consequence for their academic achievement and later success in life. In Brazil, approximately one-third of third graders are not able to read more than isolated words and phrases or find specific information in text (PREAL, 2009). A better understanding of the cognitive profile of children with low reading achievement in the classroom is thus crucial for the early identification of children at risk of academic failure and to improve educational outcomes for disadvantaged children.

In summary, the purpose of this study is twofold. Firstly to explore the extent to which different executive function components relate to reading achievement as measured by teacher evaluation in the early school grades. Secondly, to shed light on the executive function profile that accompanies low reading achievement in general education classrooms in Brazil. Research considering various components of executive functioning in a single study in young children is limited. This is particularly true for children at increased risk of academic failure such as those from low-income homes who are often excluded from scientific studies. Our study addresses the following questions:

1. Do separable executive function components make differential contributions to reading achievement in early readers from Brazil?
2. Do Brazilian children whose classroom reading performance in decoding and reading comprehension is judged below standard by their teachers differ from children with average or good reading scores on measures of executive functioning?
3. What executive functioning components separate the performance of the low reading achievers from those with average or good reading scores and what is the predictive capacity of the identified components for classifying students as good or poor readers?

## Materials and methods

### Sampling procedure

Children were recruited from public (i.e., state) and private schools across two Brazilian states—Bahia (BA, Northeast) and São Paulo (SP, Southeast). A range of schools from neighborhoods of different socioeconomic status levels in the cities of Salvador (BA) and São Paulo (SP) were contacted. We avoided schools that were located in extremely poor or dangerous neighborhoods, charged very high school fees or were bilingual. In total, 17 primary schools (53 classrooms) agreed to participate, of which 11 were located in Salvador and 6 in São Paulo. The data was collected as part of a larger study on the effects of poverty on children's cognitive development. At the time the study was conducted, children in Brazil started their reading instruction in Year 1 of primary, when they were around 6 years of age.

Caregivers of children from 1° *Ano* (Year 1) and 2° *Ano* (Year 2) of the *Ensino Fundamental I* (primary education I) of the selected schools were invited to complete the *Questionário Brasileiro do Ambiente Infantil* (QBAI, Brazilian Questionnaire of Children's Background) that was designed for this study. It contains questions pertaining to early childhood experiences, information on the medical and developmental history of the child and demographic and socio-economic characteristics of the household. The nutritional status of each child was assessed using anthropometric measurements (height, weight, and mid-upper arm circumference) following the recommendations of the World Health Organization (2007). Children also completed a non-verbal reasoning/IQ test (Raven Progressive Colored Matrices, Raven et al., 1986). Exclusion criteria for participation in the study were: maternal alcohol or drug use reported during pregnancy; severe complications at pregnancy or birth; very premature births (less than 32 weeks of gestation) or very low birth weight (1500 g or less); neurological impairments, history of head injury or other significant medical problems; moderate or severe stunted growth (below -2 SD from median height-for-age of reference population); moderate or severe wasting (below -2 SD from median weight-for-height of reference population); developmental delays or intellectual disability; learning disorder; victims of abuse; scholarship holders (*bolsistas*); bilingualism and chronological age outside the expected range.

In total 482 caregivers were interviewed, of whom approximately half were sending their children to private schools. 82 children were not tested because they did not meet the inclusion criteria and 5 children were excluded due to missing data. Complete data was obtained on 395 children. Our aim was to recruit a sample of typically developing children. The developmental and medical history of a subsample of children had to be assessed further by a team of physicians and for some children missing background information had to be completed by additional interviews. This led to a further exclusion of 40 participants for the following reasons: significant medical concerns (e.g., low APGAR scores; eclampsia, *N* = 13); maternal alcohol or drug abuse during pregnancy (*N* = 9); very premature or very low birth weight (*N* = 4); undernutrition (*N* = 2); Raven's score below the 5th percentile (*N* = 4); learning disorder or significant sensory impairment (*N* = 4); victim of abuse or domestic violence (*N* = 4).

Three hundred and fifty-five participants fulfilled all inclusion criteria. Teachers of these children were asked to rate each child's word decoding and reading comprehension achievement on a scale from 0 (very bad) to 10 (very good). This format corresponds to the standard grading scale used in Brazilian schools. From this sample, children with scores at or below 5 in both word decoding and reading comprehension were selected. Our cutoff score for determining whether a child is a “poor reader” is based on common educational practice in Brazil where a score of 6 (sometimes 5) is generally considered the minimum passing grade. The number of children identified as poor readers (from a total *N* = 355) was 53 (13%). These poor readers were matched for chronological age, sex, school type (private or public), domicile (Salvador or São Paulo) and socioeconomic status with 53 children presenting satisfactory reading scores of 6 or above in both decoding and reading comprehension. For simplicity, the latter group is referred to as the group of “good readers.”

### Participants

Descriptive characteristics of the groups are reported in Table 1. All children lived in an urban setting, were monolingual in Portuguese, and had a mean age of 7 years and 6 months (ranging from 6 years and 4 months to 8 years and 10 months) with no significant difference in age [*t*_(104)_ = 1.28; *p* = 0.20] among the two groups. The information obtained from the QBAI allowed us to calculate for each child the score on the *Critério de Classificação Econômica Brasil* (CCEB, Brazilian Criteria for Economic Classification, ABEP, 2010). The CCEB is commonly used in Brazil to segment the population into different economic classes (eight classes ranging from A1=very high socioeconomic status to E=very low socioeconomic status). We also computed for each child the score on the International Socio-Economic Index of Occupational Status (ISEI; Ganzeboom, 2010). The index is based on a meta-analysis by Ganzeboom et al. (1992) and was designed to capture the attributes of occupations that convert caregivers' education into income. The score can range from 16 (e.g., cleaner) to 90 (e.g., judge). The index was derived from caregiver responses on caregiver occupation and was based on the highest occupational level of either caregiver.

**Table 1:** **Descriptive characteristics of the sample according to group**.

**Measures**	**Poor readers (*N* = 53)**				**Good readers (*N* = 53)**				**Significance**
	**Freq**.	**Mean**	***SD***	**Range**	**Freq**.	**Mean**	***SD***	**Range**	***p* level**
Age (in months)	–	91.11	7.74	76–106	–	89.19	7.72	77–105	n.s.
Sex (% boys)	56.60	–	–	–	56.60	–	–	–	n.s.
School type (% public)	83.00	–	–	–	83.00	–	–	–	n.s.
City (% Salvador/BA)	52.80	–	–	–	52.80	–	–	–	n.s.
Economic class (CCEB)									
A1	3.80	–	–	–	3.80	–	–	–	n.s.
A2	7.50	–	–	–	7.50	–	–	–	n.s.
B1	5.70	–	–	–	5.70	–	–	–	n.s.
B2	13.20	–	–	–	13.20	–	–	–	n.s.
C1	26.40	–	–	–	26.40	–	–	–	n.s.
C2	34.00	–	–	–	34.00	–	–	–	n.s.
D	9.40	–	–	–	9.40	–	–	–	n.s.
International Socioeconomic Status Index		38.00	16.00	17–89		40.26	17.58	17–85	n.s.
Lenght of schooling (in months)		40.36	14.35	6–68		39.32	13.01	8–57	n.s.
Non-verbal reasoning (Raven, percentile)		46.49	23.13	11–99		60.24	25.06	11–99	<0.05
Academic achievement (out of 10)									
Decoding		3.85	1.31	1–5		8.21	1.42	6–10	<0.001
Reading compr.		3.83	1.20	1–5		8.19	1.49	6–10	<0.001
Writing		4.19	1.33	1–6.7		7.67	1.61	4–10	<0.001
Mathematics		4.62	1.66	1–10		8.12	1.57	4–10	<0.001
Oral language		5.24	1.41	1–9		8.34	1.53	5–10	<0.001
Science		5.36	1.77	1–10		8.26	1.36	5–10	<0.001
Composite		4.86	1.35	1–8		8.16	1.32	5–10	<0.001

*CCEB: Critério de Classificação Econômica Brasil (Brazilian Criteria for Economic Classification, ABEP, 2010); Raven: Raven Colored Progressive Matrices. Reading compr: reading comprehension*.

Key sample demographics were as follows: 57% were boys, 83% were frequenting public schools (free of charge), 53% lived in Salvador, and the majority (60%) of the sample fell in the social class C corresponding to gross mean household incomes between R$ 933.00—1391.00 (~US$ 393.00—585.00; ABEP, 2010). Groups were matched on these demographic variables; ratios across the two groups were therefore identical. No significant group differences emerged in terms of length of schooling [*t*_(104)_ = 0.39; *p* = 0.70] and the International Socioeconomic Status Index [*t*_(104)_ = 0.69; *p* = 0.49]. Approximately half of the children in each group (52% of the poor readers, 43% of the good readers) lived below the poverty line that was set at 50% of the median disposable income in Brazil (OECD, 2011). The sample was ethnically diverse: 50% of the children were multiracial, 25% were black and 25% were white. The good readers outperformed the poor readers on the measure of non-verbal reasoning [Raven: *t*_(104)_ = 2.94; *p* < 0.05].

As expected, significant group effects emerged for the classification measures decoding [*t*_(104)_ = 16.45, *p* < 0.001] and reading comprehension [*t*_(104)_ = 16.53, *p* < 0.001]. Significant group effects in favor of the good reading group also emerged on the non-classification measures of writing [*t*_(104)_ = 12.14, *p* < 0.001], mathematics [*t*_(104)_ = 11.12, *p* < 0.001], oral language [*t*_(104)_ = 10.82, *p* < 0.001], science [*t*_(104)_ = 9.45, *p* < 0.001], and on the scholastic achievement composite score [*t*_(104)_ = 12.70, *p* < 0.001]. Importantly, 85% of the poor readers achieved low writing scores (at or below 5); 72% had achieved failing mathematics scores, 59% were struggling with their oral language skills and 64% had difficulties in science. In the group of good readers percentages of children with scores at or below 5 were as follows: 9% for writing, 4% for mathematics, 2% for oral language and 2% for science.

### Task development

In Brazil, standardized tests that can be used to assess executive functioning in young children are scarce. The authors reviewed critically a large number of national and international instruments and discussed them with an expert panel composed of researchers, psychologists and teachers. Task selection was theory-driven. The material was carefully adapted or developed for the Brazilian context, and piloted on a Brazilian population. Task instructions from published English tests were translated into Brazilian Portuguese by a member of the research team (CJT) who is a native Brazilian and fluent in English. The translations together with the English originals were then revised by an expert panel of five independent assessors fluent in both Portuguese and English and the best features of the different revisions were retained. The measures were pre-tested and problematic items were further modified by the expert panel including the original translator. Reliability of instruments was established and is reported in the result section. A summary of the executive function tests that were used and the hypothesized executive function component that they relate to are listed in Table 2 and are described in detail below.

**Table 2:** **Executive function measures selected for this study**.

**Hypothesized executive function component**	**Tasks**
Cognitive flexibility	Duck task modified from the Dimensional Change Card Sort (Zelazo, 2006)
	Opposite worlds task from the Test of Everyday Attention for Children (Manly et al., 1998)
Working memory	Digit recall task of the Automated Working Memory Assessment (Alloway, 2007)
	Counting recall task of the Automated Working Memory Assessment (Alloway, 2007)
	Dot matrix task of the Automated Working Memory Assessment (Alloway, 2007)
	Odd-one-out task of the Automated Working Memory Assessment (Alloway, 2007)
Inhibition	O Mestre Mandou (“Simon says”)
	Go/No-Go modified from Cragg and Nation (2008)
	Simon task
	Flanker task modified from the Attention Network Task from Rueda et al. (2004)
Selective attention	Map mission from the Test of Everyday Attention for Children (Manly et al., 1998)
	Sky search from the Test of Everyday Attention for Children (Manly et al., 1998)

### Procedure

Informed written consent procedures were followed for all participants and the study was approved by the ethics committees of the University of Luxembourg and the Federal University of São Paulo, the *Hospital das Clínicas* of the School of Medicine of the University of São Paulo, the *Maternidade Climério de Oliveira* of the Federal University of Bahia, as well as the national Brazilian ethics committee *Comissão Nacional de Ética em Pesquisa* (CONEP, National Commission of Ethics in Research). Each child was assessed individually in a calm area of the school in two sessions that took place on different days and that lasted approximately 1 h each. Short breaks were used within sessions to maintain motivation. The measures were administered in a fixed sequence designed to vary the nature of the task demands across successive tests. Children received a sticker after completing different phases of the assessment and a diploma for their participation at the end of testing. They were tested by 8 research assistants who had all been trained by a member of the research team (PEdA). In total, children completed a battery of 19 tasks tapping executive functioning and other cognitive domains; the results on the 12 executive function tasks are reported here. Executive functioning was assessed with paper-and-pencil or computerized tasks.

For all measures, scores were converted to T-scores using the sample mean and standard deviation from the complete sample of 355 Brazilian children as a reference. The signs of the scores of variables on which low scores indicate better performance were inverted so that all positive scores represent superior performance.

### Measures

#### Non-verbal reasoning

Children completed the *Raven Colored Progressive Matrices Test* (Raven et al., 1986) in which they have to complete a geometrical figure by choosing the missing piece among 6 possible drawings. Patterns increase progressively in difficulty and the test consisted of 36 items. Norms on a population of Brazilian children are available for this test (Angelini et al., 1999).

#### Cognitive flexibility

Two cognitive flexibility measures were administered: the *Duck Task* and the *Opposite Worlds* task. Both tasks contain different conditions and children have to switch from one condition to the other.

The *Duck Task* is a dimensional change card-sorting task that was modified from Zelazo (2006). Children have to sort bivalent test cards (red/blue; duck/flower) according to one specific rule (color or shape). The sorting rule changes across the task but the stimuli cards remain the same with each card representing the two dimensions at the same time. Two target cards (a blue duck and a red flower) are attached to sorting trays and remain visible throughout the task. Cards have to be placed facedown in one of the trays. Children are first told to sort the cards by shape (“shape game”) and then by color (“color game”). In each case the experimenter explains the sorting rule and demonstrates two examples. The child then completes two practice trials with feedback followed by six experimental trials without feedback. In the next task, cards that contain an additional star sticker are introduced. Children are told that the star sticker cards need to be sorted by color whereas the cards without a star have to be sorted by shape (“shape-color game”). The experimenter demonstrates two examples (one with a star) and verifies verbally if the child understood the rules of the game. Children then complete two practice trials with feedback (one with a star). If the practice trials are failed the experimenter repeats the rules of the game and the child completes two further practice items with feedback. After these practice trials, the children are reminded of the rules of the game and then the experimental trials start. Children have to sort 24 cards with a rule reminder after 12 trials but no feedback. The majority of the cards (16) have to be sorted by shape; one-third of the trials (8) are star sticker trials. On the “shape game” and “color game” children scored at ceiling. The number of correctly sorted star sticker trials on the “shape-color game” was used as dependent variable in the analyses.

The *Opposite Worlds* task is part of the *Test of Everyday Attention for Children* (Manly et al., 1998). Children are presented with stimulus sheets containing each a weaving path of the digits one and two. In the “same world” condition they have to follow the path and name the digits as quickly as possible in the conventional manner. In the “opposite world” condition they are asked to say “two” for the digit one and “one” for the digit two as they proceed along the path. The task starts with the “same world” condition, followed by two “opposite world” conditions and a final “same world” condition. The dependent variable used for these analyses was the sum of correct responses.

#### Working memory

Working memory was assessed with four sub-tests from the computer-based *Automated Working Memory Assessment* (AWMA, Alloway, 2007). The measures are verbal or visuo-spatial span tasks in which the number of items to be remembered increases progressively over successive blocks that contain six trials each. Testing stops after three errors in one block and the number of correctly recalled trials serves as the dependent variable.

Two verbal working memory measures—*Digit Recall* and *Counting Recall*—were administered. *Digit Recall* is a simple span task in which children have to repeat immediately sequences of spoken digits in the order that they were presented. In the *Counting Recall* task (a complex span task) children have to count and memorize the number of circles in pictures containing circles and triangles. At the end of each trial the number of circles in each picture has to be recalled in the right order.

Children also completed two measures of visuo-spatial working memory: The *Dot Matrix* and the *Odd-One-Out* tasks. In the simple span task *Dot Matrix*, children see a 4 × 4 matrix and a red dot that appears in different locations on the matrix. Children have to remember the sequence of the locations and recall them by tapping the squares of the empty matrix in the right order at the end of each trial. The *Odd-One-Out* task is a complex span task in which children are presented with arrays of three boxes with one shape in each. Two shapes are identical. Children have to identify the non-matching shape, remember its location in each array, and recall the localization of the odd shape when presented with an array of empty boxes at the end of the trial.

#### Inhibition

Response inhibition was assessed with two tasks (“*O Mestre Mandou*” and a *Go/No-Go* task) in which only certain conditions require a motor response while others must be ignored. Children also completed a *Simon* and a *Flanker* task of interference suppression. In these tasks, the features of bivalent displays either converge on a single response (creating congruent trials) or conflict by indicating different responses (creating incongruent trials).

In the “*O Mestre Mandou*” (“the master ordered”) task, a Brazilian version of the children's game “Simon says,” children stand opposite the experimenter who performs a series of physical actions accompanied by verbal commands (e.g., “touch your head”). Children have to imitate the actions of the experimenter if the command is prefaced with the phrase “*o mestre mandou*” but they must stand still for commands that do not begin with “*o mestre mandou*.” The experimenter performs all the actions irrespective of the instruction. In total, 16 trials are administered, of which 8 are non-imitation trials. The task is preceded by two practice trials with corrective feedback and children are reminded of the task rules after the first half of test trials. The dependent measure used for analyses is the sum of responses on the non-imitation trials that are coded as: 3 for no movement, 2 for wrong movement, 1 for partial imitation, and 0 for complete imitation.

The *Go/No-Go* task used was an adapted version of an English task by Cragg and Nation (2008). The task is presented on a laptop computer and consists of a background scene of a soccer goal and either a soccer ball (Go trials) or an American football ball (No-Go trials) that appears for 200 ms centrally near the bottom of the screen. Children are instructed to continuously press down the left mouse button (marked with a star) with the index finger of their dominant hand. When the soccer ball appears they are told that they have to shoot it by letting go of the star key and pressing the right mouse button as fast as possible with the same finger. When an American football ball appears they are told to keep their finger pressed down on the star key in order not to shoot the “funny looking” ball. Children first complete two blocks of 10 Go trials each. Next two mixed blocks (containing Go and No-Go trials) of 32 trials each are presented. No-Go stimuli occur on 25% of the trials. The dependent measure used for analyses was the percentage of correct responses in the mixed blocks. Go trials were scored as correct if the child released the star key and pressed the adjacent response key. No-go trials were scored as correct if the child continued pressing the star key.

The *Simon* and *Flanker* tasks were computer administered on a laptop. They were programmed and ran using the E-Prime 2.0 software (Psychology Software Tools, Pittsburgh, PA). Responses were recorded with two round colored response buttons (diameter of 2.5″), which were placed on the left and the right side next to the laptop keyboard. In the *Simon* task, green and yellow teddy bears (2.75″ × 2.56″) appear on the left and the right side of the screen. Children have to press as quickly as they can the green response button if the teddy bear is green and the yellow button if the teddy bear is yellow. Half the trials are incongruent, so the colored teddy bear appears on the side opposite to the appropriate response button. The *Flanker* task was an adapted version of the Attention Network Task by Rueda et al. (2004). A horizontal row of five equally spaced yellow fish is presented (3.35″ × 0.39″) and children have to indicate the direction of the central fish “Nemo” by pressing the corresponding left or right response buttons as fast as possible. On congruent trials (50% of all trials), the flanking fish are pointing in the same direction as the target, and on incongruent trials (50% of all trials), the distracters point in the opposite direction.

In both tasks, Simon and Flanker trials start with a fixation cross that appears in the middle of the screen for 1 s, followed by the stimulus for 5 s or until a response is made. Responses are followed by feedback and a 400-ms blank interval. Two blocks of 20 trials each have to be completed in which presentation of congruent and incongruent trials is randomized. Eight practice trials precede the experimental trials. If more than two errors occur on these trials, the instructions and the practice are repeated until the child reaches the criterion level. The dependent measures used for analyses were the reaction times (RTs) on incongruent trials (excluding incorrect responses, RTs below 200 ms and RTs above 3 *SD* of individual means).

#### Selective attention

Two timed visual search tasks from the *Test of Everyday Attention for Children* (Manly et al., 1998) were administered: *Map Mission* and *Sky Search*.

In the *Map Mission* task children are presented with an A3 size city map with various distracters (e.g., small symbols of supermarket trolleys, cars…). They have to circle as many targets (small symbols of petrol stations) as possible within 1 min with a marker pen. In total 80 targets are presented. The dependent variable is the number of targets circled.

In the *Sky Search* task, children are given an A3-sheet with 128 paired spacecrafts of which 20 pairs are identical. They have to circle the identical pairs as quickly as possible with a marker pen. Next the children complete a motor control version of the task containing only the 20 target items. For both versions of the task, children have to mark a completion box when they are finished and timing is stopped. The motor control time-per-target score is subtracted from the initial time-per-target score leading to a Sky Search score that is relatively free from the impact of motor speed.

#### Classroom teacher ratings

Teachers were asked to rate each child's academic achievement during the school year on a scale from 0 to 10 in the following areas: *leitura* (reading): *decodificação* (decoding) and *compreensão* (comprehension); *escrita* (writing): *ortografia* (orthography), *redação* (text production), and *caligrafia* (handwriting); *matemática* (mathematics): *numeração* (numeracy), *contas* (basic arithmetic operations) and *compreensão de problemas* (problem solving); *linguagem oral* (oral language): *expressão* (expression) and *compreensão* (understanding); *ciências humanas e da natureza* (human and natural sciences): *ciências* (natural sciences), *história* (history), and *geografia* (geography). Composite scores were computed for writing, mathematics, oral language and human/natural sciences by averaging the different sub-scores in each domain. For each student the total level of achievement was also calculated.

## Results

### Descriptive statistics and correlational analyses

The data did not present any missing values and none of the variables manifested severe departures from normality (Kline, 2005). Descriptive statistics for the non-verbal reasoning and executive function measures are provided in Table 3. Internal reliability estimates for the scores on the different measures were established for the complete sample (*N* = 355) using Cronbach's alpha. Reliability coefficients were in an acceptable range with reliability levels ranging from 0.60 to 0.93.

**Table 3:** **Descriptive statistics for non-verbal reasoning and executive function scores (*N* = 106)**.

**Measures**	**Mean**	***SD***	**Range**	**Cronbach's α**
**NON-VERBAL REASONING (PERCENTILE)**				
Raven CPM	53.37	27.98	10–99	0.82
**COGNITIVE FLEXIBILITY**				
Duck task	46.03	9.52	34.19–63.42	0.87
Opposite worlds	44.93	9.79	20.06–62.74	0.66
**WORKING MEMORY**				
Digit recall	45.42	9.84	22.01–76.81	0.93
Counting recall	45.48	9.58	30.00–72.89	0.92
Dot matrix	46.58	9.55	23.08–68.95	0.91
Odd-one-out	46.47	9.88	30.88–75.74	0.91
**INHIBITION**				
Mestre mandou	49.42	11.14	20.86–65.77	0.60
Go/No-Go	50.11	10.38	26.92–69.34	0.73
Simon task	46.94	9.09	24.82–73.22	0.83
Flanker task	47.53	10.21	27.38–74.22	0.87
**SELECTIVE ATTENTION**				
Map mission	48.29	8.43	30.19–78.78	N/A
Sky search	47.55	9.69	30.99–75.95	N/A

*Raven CPM: Raven Colored Progressive Matrices. With the exception of the Raven all scores are T scores. Cronbach's α was not computed on the timed selective attention measures*.

Zero-order correlation coefficients between age, non-verbal reasoning and the different executive function measures are reported in the upper triangle of Table 4. The lower triangle shows partial correlations controlling for chronological age (months). The overall pattern of relationship did not change when age was partialled out. As there exists a large overlap between fluid intelligence and executive functioning (Kyllonen and Christal, 1990; Engle et al., 1999; Conway et al., 2002; Colom et al., 2003; Kane et al., 2004; Engel de Abreu et al., 2010), non-verbal reasoning was not used as a covariate when exploring the relationship between the executive function components (Dennis et al., 2009).

**Table 4:** **Correlations between age, non-verbal reasoning and executive functioning using Pearson's correlation coefficients (*N* = 106)**.

**Measures**	**1**	**2**	**3**	**4**	**5**	**6**	**7**	**8**	**9**	**10**	**11**	**12**	**13**	**14**
1. Age	–	–	–	–	–	–	–	–	–	–	–	–	–	–
**NON-VERBAL REASONING**														
2. Raven CPM	−0.13	–	**0.35**	**0.34**	**0.43**	**0.44**	**0.47**	**0.45**	**0.26**	0.10	**0.40**	**0.36**	**0.35**	**0.26**
**COGNITIVE FLEXIBILITY**														
3. Duck task	−0.05	**0.35**	–	**0.23**	**0.26**	**0.35**	**0.26**	**0.30**	0.14	−0.07	0.13	0.04	0.02	0.09
4. Opposite worlds	0.04	**0.33**	**0.23**	–	**0.25**	**0.29**	**0.36**	**0.38**	**0.25**	**0.19**	**0.28**	0.17	**0.23**	**0.28**
**WORKING MEMORY**														
5. Digit recall	0.10	**0.41**	**0.26**	**0.26**	–	**0.52**	**0.39**	**0.38**	**0.31**	0.14	**0.23**	0.18	**0.25**	**0.22**
6. Counting recall	0.17	**0.41**	**0.34**	**0.29**	**0.53**	–	**0.38**	**0.49**	**0.25**	0.03	**0.28**	**0.25**	**0.23**	**0.33**
7. Dot matrix	**0.21**	**0.43**	**0.24**	**0.35**	**0.39**	**0.40**	–	**0.50**	**0.19**	0.18	**0.39**	**0.30**	**0.20**	0.10
8. Odd-one-out	**0.31**	**0.38**	**0.27**	**0.37**	**0.38**	**0.52**	**0.53**	–	**0.26**	0.03	**0.43**	**0.45**	0.11	0.20
**INHIBITION**														
9. Mestre mandou	0.10	**0.24**	0.14	**0.25**	**0.31**	**0.26**	**0.21**	**0.28**	–	**0.23**	0.17	**0.20**	**0.17**	0.15
10. Go/No-Go	0.17	0.10	−0.07	**0.20**	0.15	0.06	**0.21**	0.08	**0.25**	–	0.05	0.06	0.15	0.11
11. Simon task	0.17	**0.37**	0.12	**0.28**	**0.24**	**0.30**	**0.41**	**0.45**	0.18	0.07	–	**0.64**	0.17	0.22
12. Flanker task	0.10	**0.35**	0.03	0.17	0.18	**0.26**	**0.30**	**0.45**	**0.20**	0.07	**0.64**	–	**0.25**	0.19
**SELECTIVE ATTENTION**														
13. Map mission	**0.32**	**0.29**	0.00	**0.23**	**0.26**	**0.27**	**0.25**	**0.20**	0.19	**0.20**	**0.22**	**0.26**	–	**0.36**
14. Sky search	0.18	**0.24**	0.08	**0.28**	**0.23**	**0.35**	0.13	**0.24**	0.16	0.14	**0.24**	0.17	**0.39**	–

*Raven CPM: Raven Colored Progressive Matrices. Upper triangle shows first-order correlations, and lower triangle shows correlations controlling for age in months. p < 0.05 are marked in boldface*.

As expected, non-verbal reasoning was significantly related to all the executive function measures, with the exception of the Go/No-Go task (*r*'s ranging from 0.24 to 0.43). Within the areas of working memory and selective attention, measures correlated significantly with each other (*r*'s ranging from 0.38 to 0.53) and correlations demonstrating convergent validity were higher than correlations demonstrating discriminant validity. A significant correlation was also obtained between the two cognitive flexibility measures (*r* = 0.23). These tasks also manifested moderate correlations with other executive function domains, especially with working memory (*r*'s ranging between 0.26 and 0.37). For inhibition, a high correlation was obtained between the Simon and the Flanker tasks of interference suppression (*r* = 0.64) and a significant correlation emerged between the response inhibition measures Mestre mandou and Go/No-Go (*r* = 0.25). Notably, inter-correlations between measures of interference suppression and response inhibition were low (*r*'s ranging from 0.07 to 0.20). Across executive function constructs, the working memory measures manifested the highest correlations, with the other executive function domains and the response inhibition measures the lowest.

### Components of executive functioning

The 12 executive function tasks were submitted to a principal component analysis with varimax rotation of the factor structure. Four factors with eigenvalues above 1.00 were extracted, which accounted for 62.5% of the total variance. Factor loadings on the rotated matrix are listed in Table 5. A loading above 0.40 was used as a criterion for interpreting the factors. The working memory and cognitive flexibility measures loaded highly on Factor 1 (32.7%, factor loadings between 0.41 and 0.78). The interference suppression measures (Simon and Flanker) loaded on Factor 2 (10.8%, factor loadings of 0.85 and 0.87) with an additional moderate loading of the visuo-spatial working memory tasks (factor loadings of 0.46 and 0.57). Factor 3 (10.5%) included the sub-tests of selective attention (factor loadings of 0.75 and 0.83). The response inhibition measures loaded highly on Factor 4 (8.5%, factor loadings of 0.61 and 0.85). Notably, only the visuo-spatial working memory measures had loadings over 0.40 for more than one factor.

**Table 5:** **Factor loadings from principal component analysis**.

**Measures**	**Factor 1**	**Factor 2**	**Factor 3**	**Factor 4**
	***“Working Memory/Cognitive Flexibility”***	***“Interference Suppression”***	***“Selective Attention”***	***“Response Inhibition”***
Duck task	**0.78**	−0.04	−0.11	−0.10
Opposite worlds	**0.41**	0.19	0.21	0.37
Digit recall	**0.62**	0.11	0.24	0.28
Counting recall	**0.70**	0.22	0.35	0.05
Dot matrix	**0.47**	**0.46**	0.00	0.35
Odd-one-out	**0.54**	**0.57**	0.10	0.15
Mestre mandou	0.27	0.11	0.06	**0.61**
Go/No-Go	−0.12	0.00	0.12	**0.85**
Simon task	0.13	**0.85**	0.14	0.05
Flanker task	0.00	**0.87**	0.14	0.05
Map mission	0.03	0.18	**0.75**	0.21
Sky search	0.18	0.07	**0.83**	0.02

*Factor loadings above 0.40 are marked in boldface*.

The extracted four components were labeled “Working Memory/Cognitive Flexibility” (Factor 1), “Interference Suppression” (Factor 2), “Selective Attention” (Factor 3), and “Response Inhibition” (Factor 4). For each participant factor scores produced by this solution were computed using the regression method and they were used as dependent measures for the subsequent analyses.

### Relationship between executive functioning components and teacher ratings

Table 6 represents the partial correlation coefficients controlling for chronological age between the identified executive function factor structure and the different teacher ratings. The academic achievement ratings correlated strongly with each other (*r*'s ranging from 0.80 to 0.98). Correlations between the decoding and reading comprehension ratings were high (*r* = 0.98).

**Table 6:** **Partial correlations (controlling for age in months) between the identified executive function factor structure and teacher ratings using Pearson's correlation coefficients (*N* = 106)**.

**Teacher ratings**	**Factor 1**	**Factor 2**	**Factor 3**	**Factor 4**	**1**	**2**	**3**	**4**	**5**	**6**	**7**
1. Decoding	0.35^*^	**0.20**	**0.22**	**0.22**	–						
2. Reading compr.	0.36^*^	**0.20**	0.25^*^	0.18	**0.98**	**–**					
3. Writing	0.29^*^	**0.25**	0.18	**0.22**	**0.86**	**0.90**	**–**				
4. Mathematics	0.43^*^	0.29^*^	0.18	0.14	**0.88**	**0.87**	**0.86**	**–**			
5. Oral language	0.42^*^	0.18	**0.25**	0.13	**0.83**	**0.85**	**0.80**	**0.85**	**–**		
6. Sciences	0.40^*^	0.19	0.19	0.17	**0.80**	**0.82**	**0.82**	**0.87**	**0.88**	**–**	
7. Composite	0.41^*^	**0.23**	**0.22**	0.18	**0.91**	**0.92**	**0.91**	**0.94**	**0.93**	**0.96**	**–**

*Factor 1, “Working Memory/Cognitive Flexibility”; Factor 2, “Interference Suppression”; Factor 3, “Selective Attention”; Factor 4, “Response Inhibition”; Reading compr: reading comprehension. p < 0.05 are marked in boldface*.

* *Correlation coefficients that remain significant after controlling for non-verbal reasoning*.

Factor 1 correlated moderately to largely with all the teacher ratings of achievement (*r*'s ranging from 0.29 to 0.43). Factor 2 was significantly related to reading, writing and mathematics (*r*'s ranging from 0.20 to 0.29) and Factor 3 was linked significantly to ratings in reading (*r*'s of 0.22 and 0.25) and oral language (*r* of 0.25). Weak associations emerged between Factor 4 and ratings of decoding and writing (*r*'s of 0.22).

Considering the reading achievement ratings, the strongest correlations emerged with Factor 1 (*r*'s of 0.35 and 0.36). These links were notably larger than the links for reading with the other executive function factors (*r*'s ranging from 0.18 to 0.25) and remained significant even after controlling for non-verbal reasoning (*r*'s ranging from 0.21 to 0.34).

### Performance of the good and poor readers on executive functioning components

A series of Analyses of Covariance were conducted with the executive function factor scores as dependent variables. After controlling for chronological age, significant group differences emerged on the Working Memory/Cognitive Flexibility factor [*F*_(1, 103)_ = 9.29; *p* < 0.01] with good readers outperforming poor readers (poor readers: *M* = −0.28, *SD* = 1.01; good readers: *M* = 0.28, *SD* = 0.91). This group effect remained significant even after controlling for non-verbal reasoning [*F*_(1, 102)_ = 4.05; *p* < 0.05]. The groups' performance was equivalently on the remaining factors.

A logistic regression analysis was conducted to predict reading group membership using Working Memory/Cognitive Flexibility as a predictor. A test of the full model against a constant-only model was statistically significant, indicating that the Working Memory/Cognitive Flexibility factor distinguished reliably between good and poor readers [χ^2^_(1)_ = 8.64; *p* < 0.01]. Prediction success overall was 61.3%, with 66% correctly classified for the group of poor readers and 57% for the group of good readers. The Wald criterion demonstrated that Working Memory/Cognitive Flexibility made a significant contribution to prediction (*p* < 0.01).

## Discussion

This research examined executive functioning and reading achievement in 6- to 8-year-old Brazilian children. Particular strengths of the study include the heterogeneity of the population sampled (drawn from a full range of socioeconomic backgrounds and reading achievement), the use of multiple measures tapping into different executive functioning components and the thorough group matching of participants on key socio-demographic factors. Findings showed, firstly, that in this population of children, individual differences in executive functioning components make differential contributions to early reading achievement. Secondly, that children whose classroom reading performance is judged below standard by their teachers demonstrate limitations in working memory/cognitive flexibility compared to more skilled readers.

The distinction between different executive function components fits well with findings from previous studies on adults (Robbins, 1996; Miyake et al., 2000; Friedman et al., 2008) and children (Lehto et al., 2003; Senn et al., 2004; Huizinga et al., 2006; St. Clair-Thompson and Gathercole, 2006; Van der Sluis et al., 2007) and is consistent with the multicomponential framework of executive functioning (Miyake et al., 2000). In this sample of young children from Brazil, the following four executive function components were identified: (1) Working Memory/Cognitive Flexibility, (2) Interference Suppression, (3) Selective Attention, and (4) Response Inhibition. Notably, measures of cognitive flexibility did not relate to a distinguishable underlying executive function construct but instead shared a common association with the working memory measures. This finding stands in contrast to studies on adults (Miyake et al., 2000) but is in line with other research on children, indicating that cognitive flexibility may be less differentiated from working memory in young children than in older children or adults (Senn et al., 2004; St. Clair-Thompson and Gathercole, 2006). It is worth noting that cognitive flexibility might well exist as a latent construct but might be difficult to identify in exploratory factor analyses because it might not account for a large amount of variance that is not shared with measures of working memory.

Another unexpected finding was that tasks of interference suppression and response inhibition were unrelated, indicating that these measures capture distinct aspects of inhibitory control. This extends previous evidence from Martin-Rhee and Bialystok (2008) and is consistent with the view that there are several distinguishable inhibitory components (Barkley, 1997; Nigg, 2000; Friedman and Miyake, 2004). Further, results showed that verbal and visuo-spatial working memory tasks as well as simple span (i.e., short-term memory) and complex span tasks of working memory related to the same underlying factor. This demonstrate that these measures rely, in part at least, on domain-general executive resources in young children. Visuo-spatial working memory tasks were additionally linked to measures of interference suppression. This finding fits well with the theoretical account on adults that the ability to deal with interference or conflict represents one key component of working memory capacity (Oberauer and Kliegl, 2001; Braver et al., 2007; Hasher et al., 2007; Kane et al., 2007; Unsworth and Engle, 2007).

The results are consistent with previous research from English-speaking countries on independent contributions of discrete executive function components to children's academic achievement (St. Clair-Thompson and Gathercole, 2006), and extends those findings to a population of children from Brazil. The Working Memory/Cognitive Flexibility factor emerged as the best predictor of reading achievement and the magnitude of this relationship was considerably higher than the associations found between reading and other executive function components. It is notable that Working Memory/Cognitive Flexibility remained closely associated with the reading scores even when non-verbal reasoning was controlled. This result validates the account that working memory capacity provides a crucial building block for the development of early literacy skills (Swanson and Sachse-Lee, 2001; Gathercole et al., 2006a,b; St. Clair-Thompson and Gathercole, 2006; Welsh et al., 2010; Swanson et al., 2011) and shows that this relationship holds in early readers of Portuguese from Brazil.

Working memory/cognitive flexibility was also closely related to achievement in other academic domains, particularly mathematics. This finding is consistent with the view that working memory acts as a bottleneck for learning in that it supports general academic progress rather than the acquisition of skills and knowledge in specific domains (St. Clair-Thompson and Gathercole, 2006). According to Swanson and colleagues (Swanson and Saez, 2003; Swanson and Beebe-Frankenberger, 2004), working memory and scholastic achievement are related because greater working memory resources facilitate active maintainance of information and the integration of this with recent input and past knowledge. These represent key processes in academic learning. A related suggestion is that many classroom situations place heavy demands on the working memory system because children are required frequently to hold information in mind while engaging in effortful activities. Lengthy and complex classroom instructions or difficult task structures can lead to working memory overload in children with poor working memory function. This can result in task failure or abandonment, in other words, missed learning opportunities that negatively affect normal rates of learning (Gathercole et al., 2006b; Gathercole and Alloway, 2008).

Our study adds to existing evidence that struggling readers frequently display weaknesses in specific components of executive functioning. Compared to the good readers, children in the poor reading group had significantly lower scores on the Working Memory/Cognitive Flexibility factor. Unlike other authors, we did not find significant group differences on other executive function components (Reiter et al., 2005; Borella et al., 2010; Pimperton and Nation, 2010). The difference in findings could be due to the fact that previous studies focused almost exclusively on clinical populations of children with reading disorders such as dyslexia or specific reading comprehension difficulties. The present sample consisted of children without a diagnosed learning disability, drawn from typical classrooms but who had obtained low reading scores from their teachers.

It is worth noting that the focus of this study was on exploring the executive function profile of children whose classroom reading performance was judged below standard by their teachers and who were therefore at increased risk of grade repetition in Brazil. An obvious limitation of the study is that teacher ratings may be biased. It would be of interest if future studies would include standardized tests of reading achievement in a longitudinal research design. This would give a fuller appreciation of the nature of the relationship between executive functioning and reading.

This theoretical study has potential implications for practice and policy making. Learning to read is more than an educational skill. Low levels of literacy skills and living in poverty create a mutually reinforcing cycle that is difficult to break. The early identification of poor readers, together with remediation programmes that attempt to close gaps in achievement, are therefore crucial in order to counteract the impact of poverty on people's lives. Our study suggests that many students in Brazil might have fallen behind in their reading and struggle academically because of working memory limitations. Therefore teachers might want to assess whether underachieving students have working memory difficulties. Learning environments that prevent the overload of working memory resources might be a promising step toward counteracting early reading difficulties and subsequent school failure. Research from the UK has identified a number of methods of how to manage cognitive loads effectively in classroom settings (Gathercole et al., 2006b; Gathercole and Alloway, 2008). It remains to be seen whether such classroom-based approaches can enhance student learning in other cultural and educational settings. New research has also focused on supporting the development of working memory skills directly through targeted training programs (see Diamond and Lee, 2011, for a review). A range of activities have now been shown to improve children's working memory and might help children with poor academic progress overcome some of their learning difficulties (Holmes et al., 2009; Loosli et al., 2012; Alloway et al., 2013).

In conclusion, our findings indicate that distinct executive function components are predictive for individual differences in reading achievement in 6- to 8-year-old children. They also corroborate the notion that deficits in working memory/cognitive flexibility might represent one contributing factor to reading difficulties in early readers from Brazil.

### Conflict of interest statement

The authors declare that the research was conducted in the absence of any commercial or financial relationships that could be construed as a potential conflict of interest.

## Supplementary material

The Portuguese version for this article can be found online at: http://www.frontiersin.org/journal/10.3389/fpsyg.2014.00550/abstract
