# Supplementary material for: Executive functioning and reading achievement in school: a study of Brazilian children assessed by their teachers as “poor readers”
Source: Front Psychol. 2014 Jun 10;5:550. doi: 10.3389/fpsyg.2014.00550 (PMC4050967; doi:10.3389/fpsyg.2014.00550)
Supplement: Supplementary file 1 [file RelatedArticle1.PDF]

frontiers in
PSYCHOLOGY

ARTIGO CIENTÍFICO
Este artigo é uma tradução de um original em inglês
Publicado em: Junho de 2014
doi: 10.3389/fpsyg.2014.00550

Funcionamento executivo e desempenho em leitura na escola: um estudo de crianças Brasileiras identificadas por seus professores como "Maus Leitores"

Pascale M. J. Engel de Abreu[1]*, Neander Abreu[2], Carolina C. Nikaedo[3], Marina L. Puglisi[4], Carlos J. Tourinho[1], Mônica C. Miranda[3], Debora M. Befi-Lopes[4], Orlando F. A. Bueno[3] and Romain Martin[1]

[1] ECCS Research Unit, University of Luxembourg, Walferdange, Luxemburgo
[2] Insituto de Psicologia, Universidade Federal da Bahia, Salvador, Brasil
[3] Departamento de Psicobiologia, Universidade Federal de São Paulo, São Paulo, Brasil
[4] Departamento de Fisioterapia Fonoaudiologia e Terapia Ocupacional, Universidade de São Paulo, São Paulo, Brasil

Editado por:
Nicolas Chevalier, University of Edinburgh, UK

Revisado por:
Caron Ann Campbell Clark, University of Oregon, USA
Helen St Clair-Thompson, University of Newcastle, UK
Hannah Pimperton, University College London, UK

****Contato:***
Pascale M. J. Engel de Abreu, ECCS Research Unit, University of Luxembourg, B.P.2, L-7201 Walferdange, Luxembourg
e-mail: pascale.engel@uni.lu

Este estudo investigou o funcionamento executivo e o desempenho em leitura de 106 crianças Brasileiras de 6 a 8 anos de uma ampla diversidade social, das quais aproximadamente metade vivia abaixo da linha da pobreza. Foi dado um enfoque especial à exploração do perfil de funcionamento executivo das crianças que apresentaram desempenho em leitura abaixo da média, de acordo com avaliação dos professores. Estas crianças foram pareadas a sujeitos controle em relação à idade, sexo, tipo de escola (privada ou pública), local de domicílio (Salvador/BA ou São Paulo/ SP) e nível socioeconômico. As crianças responderam a uma bateria de 12 tarefas de funções executivas que abordam os conceitos de flexibilidade cognitiva, memória operacional, inibição e atenção seletiva. Cada domínio executivo foi avaliado por diversas tarefas. A análise de componentes principais extraiu quatro fatores que foram classificados como "Memória operacional/ Flexibilidade cognitiva", "Supressão de interferência", "Atenção seletiva" e "Resposta inibitória". Diferenças individuais nos componentes de funcionamento executivo contribuíram de maneira diferente para o desempenho precoce em leitura. O fator Memória operacional/ Flexibilidade cognitiva foi o melhor preditor de leitura. Comparações entre grupos nos escores fatoriais demonstraram que as crianças com dificuldades de leitura apresentaram um desempenho inferior em Memória Operacional/Flexibilidade, mas não nos demais componentes das funções executivas, quando comparadas com os leitores eficientes. Esses resultados corroboram a visão de que a capacidade de memória operacional fornece a base de sustentação para o desenvolvimento de habilidades de leitura, estendendo-a à população de leitores iniciantes do português no Brasil. O estudo sugere que déficits em memória operacional/ flexibilidade cognitiva podem representar um fator que contribui para dificuldades de leitura em leitores iniciantes. Tal achado é de extrema importância para desenvolvimento de intervenção para crianças em situação de risco para fracasso escolar.

Palavras-chave: funções executivas, leitura, memória operacional, flexibilidade cognitiva, atenção seletiva, inibição, pobreza, dificuldades de aprendizagem

INTRODUÇÃO

A leitura é uma tarefa complexa que depende de uma série de habilidades. Atualmente, está claro que as habilidades de consciência fonológica, conhecimento letra-som e habilidades gerais de linguagem têm um papel fundamental sobre o desenvolvimento da leitura (Carroll, Snowling, Hulme, e Stevenson, 2003; Fricke, Bowyer-Crane, Haley, Hulme, e Snowling 2013; Muter, Hulme, Snowling, e Stevenson, 2004; Nation, Clarke, Marshall, e Durand, 2004, Nation, Cocksey, Taylor, e Bishop, 2010; Rose, 2006). Mais recentemente, as habilidades que envolvem as funções executivas têm sido apontadas como mais um componente crucial para o desenvolvimento da alfabetização[1]. Crianças que apresentam problemas para ler fluentemente, ou não compreendem bem o que leem, geralmente têm problemas com as funções executivas (Reiter, Tucha, e Lange, 2005; Sesma, Mahone, Levine,

[1] Conforme utilizado em estudos prévios Brasileiros na área da Psicologia[1,2], optamos por adotar o termo "alfabetização" em referência ao original "literacy" para refletir a maior ênfase aos aspectos relacionados à decodificação que o termo em inglês carrega. No entanto, é importante mencionar que outras traduções[3,4], principalmente na Educação e Sociologia, preferem a nomenclatura "letramento" para se referir a processos mais abrangentes envolvidos no desenvolvimento proficiente da leitura.

1. Capovilla AGS et al. Estratégias de leitura e desempenho em escrita no início da alfabetização. *Psicologia Escolar e Educacional*, 2004; 8(2): 189-197.
2. Capovilla FC. Recursos para educação de crianças com necessidades especiais e articulação entre educação especial e inclusiva. *O Mundo da Saúde São Paulo*, 2008; 32(2): 208-214.
3. Soares M. Letramento: um tema em três gêneros. Belo Horizonte: Autêntica, 1998.
4. Soares M. Alfabetização e letramento. São Paulo: Contexto, 2003.

Eason, e Cutting, 2009; Locascio, Mahone, Eason, e Cutting, 2010; Pimperton e Nation, 2010, 2014). Muito se discute, no entanto, sobre a natureza e o grau de dificuldades executivas apresentado por crianças com dificuldades de leitura.

O termo "funções executivas" compreende uma série de processos cognitivos utilizados para coordenar e controlar pensamentos e ações, particularmente em novos contextos, e são cruciais para a resolução de problemas de alta complexidade e comportamento direcionado a uma meta (Zelazo e Carlson, 2012; Zelazo, Carlson e Kesek, 2008). O funcionamento executivo é geralmente avaliado por tarefas complexas, como a da Torre de Londres (*Tower of London*) ou o teste de Classificação de Cartas Wisconsin (*Wisconsin Card Sorting Test*), que envolvem várias habilidades executivas de baixa complexidade. Estudos utilizando testes complexos de funções executivas, como estes, reportam em geral correlações com a alfabetização (Hooper, Swartz, Wakely, de Kruif, e Montgomery, 2002; Sesma, et al., 2009). Modelos teóricos recentes indicam que em adultos, diferentes funções executivas consistem em componentes distintos, embora relacionados entre si (Friedman et al., 2008; Miyake et al., 2000). Também há evidências sugerindo que as funções executivas representam um conjunto de habilidades dissociáveis em crianças, embora a natureza destes fatores difira amplamente entre estudos e populações (Huizinga, Dolan, e van der Molen, 2006; Lehto, Juujärvi, Kooistra, e Pulkkinen, 2003; Rose, Feldman, e Jankowski, 2011; Senn, Espy, e Kaufmann, 2004; Steele, Cornish, Karmiloff-Smith, e Scerif, 2012; St Clair-Thompson e Gathercole, 2006; Van der Sluis, de Jong, e van der Leijs, 2007; Wiebe, Espy, e Charak, 2008). Flexibilidade cognitiva, memória operacional e controle inibitório são considerados por muitos como os componentes estruturais das funções executivas, pois são relativamente bem definidos conceitualmente, geralmente surgem como construtos distintos em modelos de análise de fatores, e têm sido relacionados ao desempenho em tarefas mais complexas de funções executivas (Baddeley, 1996; Lehto et al., 2003; Miyake et al., 2000; Rabbitt, 1997; Roberts e Pennington, 1996).

Um conceito intimamente relacionado às funções executivas é o da atenção, e várias descrições de funcionamento executivo também incluem subfunções da atenção (Breckenridge, Braddick, e Atkinson, 2013; Klenberg et al., 2001; Loher e Roebers, 2013; Manly et al., 2001). Em uma tentativa para separar os diferentes componentes das funções executivas a partir da análise fatorial exploratória em crianças de 7 a 12 anos, Klenberg e colaboradores (2001) reportaram que inibição, atenção auditiva, atenção seletiva visual e fluência constituíram fatores distintos. Em outra análise fatorial exploratória envolvendo crianças de 11 anos, foi identificada uma estrutura de dois fatores: um associado à memória operacional, e outro à inibição. O estudo também incluiu medidas de flexibilidade cognitiva, que não compuseram, no entanto, um fator executivo distinto (St Clair-Thompson e Gathercole, 2006).

A memória operacional tem sido descrita como um sistema cognitivo de múltiplos componentes, usada para armazenar e processar mentalmente as informações por breves períodos de tempo (Baddeley, 2000). A maioria dos teóricos acredita que a memória operacional reúne mecanismos relacionados à manutenção da informação por um curto período de tempo, também conhecida como memória de curto prazo, e ao processamento responsável pelo controle executivo, que regula e coordena estas operações (Engle, Tuholski, Laughlin, e Conway, 1999). Enquanto as tarefas chamadas de *span* simples cobrem principalmente o componente de armazenamento a curto prazo do sistema de memória operacional, o desempenho em tarefas de *span* complexo, que envolvem o processamento e armazenamento simultâneos da informação, parece depender tanto dos recursos executivos centrais quanto de sistemas de armazenamento de curto prazo específicos a um domínio (Duff e Logie, 2001). Alguns estudos mostram uma grande – ou até completa – sobreposição entre tarefas de memória operacional que envolvem *span* simples e complexo (Alloway, Gathercole, e Pickering, 2006), e alguns autores têm apontado que os dois tipos de medidas avaliam essencialmente o mesmo processo cognitivo subjacente (Unsworth e Engle, 2007). Há alguma evidência para componentes discretamente distintos de memória operacional verbal e visuoespacial (Shah e Miyake, 1996; Friedman e Miyake, 2000; Jarvis e Gathercole, 2003; Kane, et al. 2004). Em crianças, as tarefas de memória operacional verbal e visuoespacial podem estar relacionadas ao mesmo fator subjacente, mas ainda assim respondem por uma parcela única da variância no desempenho acadêmico (St Clair-Thompson e Gathercole, 2006). As medidas de memória operacional verbal e visuoespacial devem, portanto, refletir em parte os mecanismos de domínio geral, em parte a contribuição específica de cada modalidade dos sistemas de armazenamento (Baddeley e Logie, 1999; St Clair-Thompson e Gathercole, 2006).

Flexibilidade cognitiva (também conhecida como alternância entre tarefas) refere-se à habilidade de adaptar pensamentos ou ações de forma flexível às demandas de cada situação (Cragg e Nation, 2008). É geralmente avaliada por testes que consistem em diferentes condições, e requerem que os indivíduos alternem entre duas condições em resposta a uma pista externa. O controle inibitório denota os processos envolvidos em suprimir estímulos ou respostas preponderantes, mas irrelevantes

(Nigg, 2000). Vários subtipos de inibição foram propostos (Barkley, 1997; Friedman e Miyake, 2004; Martin-Rhee e Bialystok, 2008; Nigg, 2000). Por exemplo, Martin-Rhee e Bialystok (2008) distinguiram tarefas de inibição de resposta, que requerem o cancelamento de respostas habituais em função de aparatos univalentes (e.g. paradigma do Go/No-Go), daquelas que envolvem a supressão de interferência envolvendo aparatos bivalentes, nas quais duas características dos estímulos podem gerar respostas potencialmente conflitantes (e.g. paradigma do Flanker). A atenção seletiva refere-se à habilidade de focar em uma informação particular e ignorar estímulos irrelevantes. É geralmente medida por paradigmas de busca visual em que os objetos-alvo ou características-alvo devem ser encontradas dentre outros distratores (Manly et al., 2001; Scerif, Cornish, Wilding, Driver, e Karmiloff-Smith, 2004).

Pesquisas recentes corroboram a ideia de que o funcionamento executivo possa contribuir para o desenvolvimento da leitura. A memória operacional tem sido a mais frequentemente estudada e vários achados apontam para uma relação positiva entre o desempenho em tarefas de memória operacional e proficiência em leitura (Gathercole, Alloway, Willis, e Adams, 2006; St Clair-Thompson e Gathercole, 2006; Swanson e Sachse-Lee, 2001; Swanson, Orosco, Lussier, Gerber, e Guzman-Orth, 2011; Swanson, Saez, Gerber, e Leafstedt, 2004; Welsh, Nix, Blair, Bierman, e Nelson, 2010). Enquanto as tarefas de memória verbal de curto prazo têm sido constantemente relacionadas à habilidades de decodificação, as tarefas de *span* complexo parecem contribuir significativamente para a compreensão de leitura (Engel de Abreu e Gathercole, 2012; Swanson e Berninger, 1995). A memória operacional também tem sido relacionada a outras áreas do aprendizado acadêmico. Crianças com baixa capacidade de memória operacional têm em geral um pior progresso acadêmico, levando à hipótese de que a memória operacional deve atuar como um gargalo para a aprendizagem (Gathercole e Alloway, 2008). A flexibilidade cognitiva também foi associada à habilidade de leitura (Cartwright, 2012; Hooper et al., 2002; Welsh et al., 2010; Van der Sluis et al., 2007). Em um estudo norte-americano, Welsh e colaboradores (2010) descobriram que a habilidade de flexibilidade cognitiva em pré-escolares predisse suas habilidades de decodificação e de reconhecimento de palavras no final dos anos pré-escolares. De forma semelhante, Van der Sluis e colaboradores (2007) demonstraram que a flexibilidade cognitiva esteve positivamente relacionada à eficiência de leitura de palavras em crianças Holandesas de 9 a 12 anos.

Poucos estudos investigaram as relações entre inibição e a atenção seletiva com a leitura. Processos inibitórios mostraram implicação com a leitura em alguns estudos (De Beni e Palladino, 2000; De Beni, Palladino, Pazzaglia, e Cornoldi, 1998), mas não em outros (Lan, Legare, Ponitz, Li, e Morrison, 2011). Lan e colaboradores (2011) exploraram inibição, memória operacional e atenção seletiva de forma transcultural em crianças pré-escolares da China e dos EUA, e encontraram que a atenção seletiva foi o preditor mais robusto de identificação letra-palavra em ambos os países. No entanto, em um estudo longitudinal de crianças britânicas de 3 a 6 anos Steele e colaboradores (2012) não encontraram relação entre a habilidade de selecionar e sustentar a atenção e o reconhecimento de palavras no ano seguinte. Existe alguma evidência de que crianças com dificuldades de leitura apresentam limitações em tarefas de atenção seletiva (Romani, Tsouknida, di Betta, Olson, 2011; Sireteanu· Goebel, Goertz, Werner, Nalewajko, e Thiel, 2008). Por exemplo, Casco, Tressoldi e Dellantonio (1998) mostraram que crianças de 11 a 12 anos com o pior desempenho em uma tarefa de atenção seletiva tiveram piores notas em fluência de leitura do que crianças com as melhores habilidades de atenção seletiva.

Outra pesquisa explorando a contribuição do funcionamento executivo sobre a leitura teve como foco grupos clínicos. Reiter e colaboradores (2005) descobriram que crianças com dislexia apresentaram déficits em medidas de memória operacional verbal e visuoespacial, inibição, planejamento e flexibilidade cognitiva, quando comparados a crianças com desenvolvimento típico. Evidências crescentes também sugerem que dificuldades específicas de compreensão de leitura estão relacionadas a uma disfunção executiva (Borella, Carretti, e Pelegrina, 2010; Cain, 2006; Locascio et al., 2010; Nation, Adams, Bowyer-Crane, e Snowling, 1999; Pimperton e Nation, 2010; Sesma et al. 2009). Em um estudo dos EUA, Locascio e colaboradores (2010) identificaram que crianças com dificuldades específicas de compreensão de leitura ("leitores com pobre compreensão") tiveram desempenho abaixo do esperado em tarefas de planejamento e memória operacional visuoespacial. Achados do Reino Unido indicam, no entanto, que os leitores com pobre compreensão apresentam déficits de memória operacional e inibição que são específicos ao domínio verbal (Pimperton e Nation, 2010).

Em suma, diferenças no funcionamento executivo foram identificadas em bons e maus leitores, mas ainda permanece incerto quais componentes específicos das funções executivas estão afetados. Poucos estudos incluíram uma bateria completa de testes que abrangessem várias facetas das habilidades de funcionamento executivo. Além disso, estudos anteriores focaram quase exclusivamente em crianças falantes do Inglês dos EUA e Reino Unido. Pouco se sabe sobre as relações entre

componentes específicos das funções executivas e a leitura em outros contextos culturais e linguísticos.

O PRESENTE ESTUDO

Este estudo foi realizado no Brasil, com crianças em desenvolvimento típico dos primeiros anos do Ensino Fundamental I. No Brasil, as crianças aprendem a ler e escrever em Português, uma língua romana falada por aproximadamente 200 milhões de pessoas no mundo todo. A ortografia do Português é mais transparente que a do Inglês, embora menos transparente que outras línguas europeias como Alemão ou Italiano (Pinheiro, 1995). A despeito de grandes melhoras ao longo da última década, muitos alunos no Brasil ainda apresentam desempenho abaixo do esperado em alfabetização. Os últimos índices do "Programa Internacional de Avaliação de Alunos" (*"Programme for International Student Assessment" – PISA*), realizado pela Organização para a Cooperação e Desenvolvimento Econômico (OCDE), indicaram que o Brasil está classificado em 55 dentre 65 países no que se refere à leitura, com metade dos alunos do país apresentando desempenho abaixo do nível de proficiência básica (OCDE, 2013). O modelo construtivista representa a abordagem dominante para o ensino da leitura e escrita no Brasil (Abadzi, 2006; Belintane, 2006). Esta abordagem é baseada no princípio de que as crianças criam hipóteses sobre o código alfabético naturalmente no curso do aprendizado da leitura e escrita, e contrasta com o método fônico que é usado amplamente em países de língua Inglesa (Ehri et al., 2001; National Institute of Child Health and Human Development, 2000).

Este estudo explorou a memória operacional, flexibilidade cognitiva, inibição e atenção seletiva em uma ampla amostra de crianças pequenas provenientes de ambientes sociais diversos. Cada um destes domínios executivos foi avaliado a partir de medidas múltiplas que foram cuidadosamente selecionadas com base na literatura da Neurociência Cognitiva e são amplamente utilizadas na prática clínica e científica para medir processos relacionados ao funcionamento executivo em crianças. O objetivo foi escolher tarefas relativamente simples que mediam, do ponto de vista conceitual, componentes isolados das funções executivas. O primeiro passo para compreender a natureza da contribuição de cada um dos diferentes componentes das funções executivas para a leitura é explorar se estas funções executivas teoricamente distintas são de fato reconhecidas como fatores distintos em uma população de crianças brasileiras de uma variedade de contextos demográficos. Ressalta-se que a amostra deste estudo era etnicamente e socioeconomicamente diversa e aproximadamente 50% das crianças viviam abaixo da linha da pobreza.

O principal objetivo foi explorar o perfil de funcionamento executivo de crianças que apresentavam baixo desempenho em leitura, de acordo com a avaliação de seus professores, mas não haviam sido diagnosticadas com transtorno de aprendizagem. Há sem dúvida uma grande controvérsia com relação ao fato de professores serem ou não capazes de identificar adequadamente crianças com problemas de leitura. No entanto, em um sistema educacional como o do Brasil, o julgamento do professor sobre o desempenho da criança é fundamental, porque a repetência é uma prática comum e é basicamente realizada com base no julgamento dos professores com relação ao desempenho escolar do aluno (Bruns, Evans, e Luque, 2011). Alunos com baixo rendimento são retidos no mesmo ano escolar ao invés de progredir para o ano seguinte junto com seus colegas de mesma idade. No Brasil, quase 25% dos alunos no primeiro ano repetem um ano (PREAL, 2009), e as crianças das camadas mais pobres da sociedade são as mais afetadas (Bruns, Evans, e Luque, 2011). Os custos associados à repetência escolar no Brasil estão entre os maiores do mundo (OECD, 2011). De acordo com uma estimativa atual do Banco Mundial, o Brasil gasta aproximadamente 12% dos recursos da educação básica com repetência escolar (Bruns, Evans, e Luque, 2011). O problema da repetência escolar, no entanto, não é restrito ao Brasil; aproximadamente 32,2 milhões de crianças na educação infantil de todo o mundo repetem um ano (UNESCO, 2012). A maior razão da repetência no mundo é o baixo desempenho escolar.

Existe um consenso geral de que a leitura é uma habilidade educacional fundamental que forma a base para todos os futuros aprendizados. As crianças precisam ser capazes de ler bem para poderem engajar completamente nas atividades de sala de aula e aprender. Atualmente, muitos estudantes dos países em desenvolvimento têm dificuldades de leitura que podem ter consequências devastadoras a longo prazo para o futuro desempenho acadêmico e posterior sucesso na vida. No Brasil, aproximadamente um terço dos alunos do terceiro ano não são capazes de ler mais do que palavras isoladas e frases ou têm dificuldade de encontrar informações específicas em um texto (PREAL, 2009). Um melhor entendimento do perfil cognitivo de crianças com baixo desempenho em leitura é fundamental para a identificação precoce de crianças de risco para o fracasso escolar e para melhorar o desempenho acadêmico de crianças em situação de desigualdade.

Resumidamente, este estudo teve dois objetivos. Primeiro, explorar até que ponto os diferentes componentes das funções executivas se relacionam ao desempenho de leitura, medido pela avaliação dos professores nos primeiros anos escolares. Segundo, salientar o perfil de funcionamento executivo associado ao

baixo desempenho em leitura em sala de aula no Brasil. Pesquisas considerando vários componentes do funcionamento executivo em um único estudo são escassas. Isso é especialmente válido para crianças de alto risco para o fracasso escolar, como aquelas de baixo nível econômico que são frequentemente excluídas de estudos científicos. Este estudo investiga as seguintes questões:

1. Cada componente das funções executivas tem uma contribuição exclusiva para o desempenho acadêmico em leitores iniciais do Brasil?
2. O desempenho executivo das crianças brasileiras que apresentaram um baixo desempenho em decodificação e compreensão de leitura, de acordo com a avaliação de seus professores, difere do das crianças com leitura dentro ao acima do esperado?
3. Quais componentes do funcionamento executivo distinguem o desempenho de crianças com baixo e médio-alto desempenho em leitura? Qual é a capacidade preditiva destes componentes para classificar os alunos como bons ou maus leitores?

MATERIAIS E MÉTODOS

PROCEDIMENTO DE AMOSTRAGEM

As crianças foram recrutadas em escolas públicas e particulares de dois estados Brasileiros – Bahia (BA, Nordeste) e São Paulo (SP, Sudeste). Uma série de escolas localizadas em bairros de diferentes níveis socioeconômicos nas cidades de Salvador (BA) e São Paulo (SP) foram contatadas. Foram excluídas da amostra escolas localizadas em bairros extremamente pobres ou perigosos, escolas que cobravam mensalidades extremamente caras ou escolas bilíngues. No total, 17 escolas do Ensino Fundamental (23 turmas) concordaram em participar, sendo que 11 eram de Salvador e 6 de São Paulo. Os dados foram coletados como parte de um estudo mais abrangente sobre os efeitos da pobreza no desenvolvimento cognitivo de crianças. No momento em que o estudo foi realizado, o sistema educacional Brasileiro previa o início da instrução em leitura no Ano 1 do Ensino Fundamental I, quando as crianças têm em torno de 6 anos de idade.

Os cuidadores de crianças/ pais do Ano 1 e Ano 2 do Ensino Fundamental I das escolas selecionadas foram convidados a completar o Questionário Brasileiro do Ambiente Infantil (QBAI), que foi elaborado especialmente para este estudo. Ele contém questões relacionadas às experiências infantis, informações sobre o histórico de saúde e de desenvolvimento da criança, e características demográficas e socioeconômicas do domicílio. O nível nutricional de cada criança foi avaliado usando medidas antropométricas (altura, peso e circunferência braquial) seguindo as recomendações da Organização Mundial da Saúde (WHO, 2007). As crianças também completaram um teste de QI/ Não Verbal (Matrizes Progressivas Coloridas de Raven, Raven, Court, e Raven, 1986). Os critérios de exclusão para o estudo foram: uso de álcool ou drogas pela mãe durante a gestação; complicações severas durante a gravidez ou parto; nascimento muito prematuro (menos de 32 semanas gestacionais) ou muito baixo peso ao nascimento (1500 g ou menos); prejuízo neurológico, história de lesão cerebral ou outros problemas médicos significativos; retardo do crescimento moderado ou severo (abaixo de 2 desvios-padrão (*DP*) da mediana da altura para a idade, de acordo com a referência populacional); desnutrição moderada ou severa (abaixo de 2 *DP* da mediana do peso para a altura, de acordo com a referência populacional); atrasos do desenvolvimento ou deficiência intelectual; transtorno de aprendizagem; vítimas de abuso; bolsistas; bilinguismo e idade cronológica fora da faixa etária esperada.

No total, 482 cuidadores foram entrevistados, dos quais aproximadamente a metade tinha suas crianças matriculadas em escolas particulares. Oitenta e duas crianças não foram avaliadas porque não preenchiam os critérios de inclusão e cinco crianças foram excluídas por não terem dados completos. Foram obtidos os dados completos de 395 crianças. O objetivo foi recrutar uma amostra de crianças em desenvolvimento típico. A história médica e de desenvolvimento de uma subamostra de crianças teve de ser avaliada adicionalmente por uma equipe de médicos, e para algumas crianças, informações sobre o histórico tiveram que ser completadas em entrevistas adicionais. Isso levou à exclusão de mais 40 participantes, em virtude das seguintes razões: preocupações médicas significativas (e.g. baixo APGAR; eclampsia, N=13); uso de álcool ou drogas pela mãe durante a gravidez (N = 9); muito prematuro ou com muito baixo peso ao nascimento (N = 4); desnutrição (N = 2); desempenho no Raven abaixo do percentil 5 (N = 4); transtorno de aprendizagem ou prejuízo sensorial significativo (N = 4); vítimas de abuso ou violência doméstica (N = 4).

Trezentos e cinquenta e cinco participantes preencheram todos os critérios de inclusão. Os professores destas crianças foram solicitados a pontuar o desempenho de cada criança com relação à decodificação de palavras e compreensão de leitura em uma escala de 0 (muito ruim) a 10 (muito bom). Este formato corresponde à escala padrão de notas utilizada em escolas Brasileiras. Desta amostra, as crianças que apresentaram notas abaixo ou igual a 5 tanto na decodificação de palavras quanto na compreensão de leitura foram selecionadas. O ponto de corte utilizado para determinar se a criança seria classificada como um "mau leitor" foi baseado nas práticas educacionais usuais no Brasil, a partir das quais uma nota de 6 (às vezes até 5) é geralmente considerada a mínima para permitir a

progressão para o ano seguinte. O número de crianças identificadas como maus leitores (de um total de N = 355) foi 53 (13%). Estes maus leitores foram pareados por idade cronológica, sexo, tipo de escola (privada ou pública), domicílio (Salvador or São Paulo) e nível socioeconômico com 53 crianças que apresentaram notas satisfatórias em leitura (igual ou superior a 6) tanto em decodificação de palavras quanto em compreensão de leitura. Para simplificar, o último grupo será referido como o grupo de "bons leitores".

PARTICIPANTES

As características descritivas dos grupos são apresentadas na **Tabela 1**. Todas as crianças viviam em áreas urbanas, eram monolíngues em Português, e tinham idade média de 7 anos e 6 meses (variação de 6 anos e 4 meses a 8 anos e 10 meses), sem diferença significante de idade [$t(104) = 1.28$; $p = .20$] entre os grupos. As informações obtidas do QBAI permitiram a realização do cálculo individual da pontuação do Critério de Classificação Econômica Brasil (CCEB, ABEP, 2010). O CCEB é frequentemente utilizado no Brasil para classificar a população em diferentes classes socioeconômicas (oito classes variando de A1=altíssimo nível socioeconômico a E=baixíssimo nível socioeconômico). Também foram calculadas para cada criança a pontuação do Índice International Socioeconômico de Nível Ocupacional (*International Socio-Economic Index of Occupational Status* – ISEI; Ganzeboom, 2010). Este índice baseia-se em uma meta-análise realizada por Ganzeboom e colaboradores (1992) e foi concebido para captar os atributos das ocupações que acabam mediando o nível educacional do cuidador e o salário mensal. A pontuação pode variar de 16 (e.g. faxineiro) a 90 (e.g. juiz). O índice foi obtido a partir das respostas dos cuidadores sobre suas ocupações e foi

Tabela 1 | Características descritivas da amostra de acordo com o grupo.

Medidas	Maus leitores (N = 53)				Bons leitores (N = 53)				Significância
	Freq.	Média	*DP*	Faixa	Freq.	Média	*DP*	Faixa	Valor de *p*
Idade (meses)	--	91.11	7.74	106	--	89.19	7.72	105	n.s.
Sexo (% meninos)	56.6	--	--	--	56.6	--	--	--	n.s.
Tipo de escola (% pública)	83	--	--	--	83	--	--	--	n.s.
Cidade (% Salvador/BA)	52.8	--	--	--	52.8	--	--	--	n.s.
Classe econômica (CCEB)									
A1	3.8	--	--	--	3.8	--	--	--	n.s.
A2	7.5	--	--	--	7.5	--	--	--	n.s.
B1	5.7	--	--	--	5.7	--	--	--	n.s.
B2	13.2	--	--	--	13.2	--	--	--	n.s.
C1	26.4	--	--	--	26.4	--	--	--	n.s.
C2	34	--	--	--	34	--	--	--	n.s.
D	9.4	--	--	--	9.4	--	--	--	n.s.
Índice Internacional do Nível Socioeconômico		38	16	17-89		40.26	17.58	17-85	n.s.
Tempo de escolaridade (meses)		40.36	14.35	6-68		39.32	13.01	8-57	n.s.
Resolução não verbal (Raven, percentil)		46.49	23.13	11-99		60.24	25.06	11-99	< .05
Desempenho acadêmico (0 a 10)									
Decodificação		3.85	1.31	1-5		8.21	1.42	6-10	< .001
Compreensão de leitura		3.83	1.2	1-5		8.19	1.49	6-10	< .001
Escrita		4.19	1.33	1-6.7		7.67	1.61	4-10	< .001
Matemática		4.62	1.66	1-10		8.12	1.57	4-10	< .001
Linguagem oral		5.24	1.41	1-9		8.34	1.53	5-10	< .001
Ciências		5.36	1.77	1-10		8.26	1.36	5-10	< .001
Escore composto		4.86	1.35	1-8		8.16	1.32	5-10	< .001

CCEB: Critério de Classificação Econômica Brasil (ABEP, 2010); Raven: Matrizes Progressivas Coloridas de Raven.

baseado no maior nível ocupacional de qualquer um dos cuidadores.

As características demográficas da amostra foram as seguintes: 57% eram meninos, 83% frequentavam escolas públicas (sem mensalidade), 53% moravam em Salvador, e a maioria da amostra (60%) pertencia à classe socioeconômica C, correspondendo a um salário bruto médio de R$ 933.00 – R$ 1,391.00 (ABEP, 2010). Os grupos foram pareados de acordo com estas variáveis demográficas; as proporções entre os dois grupos foram, desta forma, idênticas. Não houve diferença estatisticamente significante entre os grupos em termos de tempo de escolaridade [$t(104) = .39$; $p = .70$] e Índice Internacional do Nível Socioeconômico [$t(104) = .69$; $p = .49$]. Aproximadamente metade das crianças de cada grupo (52% dos maus leitores, 43% dos bons leitores) viviam abaixo da linha da pobreza, caracterizada por 50% da mediana da renda líquida no Brasil (OECD, 2011). A amostra era etnicamente diversa: 50% das crianças eram multirraciais, 25% eram negros e 25% eram brancos. O desempenho dos bons leitores superou o dos maus leitores na medida de resolução não verbal [Raven: $t(104) = 2.94$; $p < .05$].

Conforme esperado, efeitos significantes entre grupos surgiram para as medidas de decodificação [$t(104) = 16.45$, $p < .001$] e compreensão de leitura [$t(104) = 16.53$, $p < .001$]. Efeitos significantes entre grupos a favor dos bons leitores também surgiram nas medidas que não foram utilizadas para classificar os grupos, como escrita [$t(104) = 12.14$, $p < .001$], matemática [$t(104) = 11.12$, $p < .001$], linguagem oral [$t(104) = 10.82$, $p < .001$], ciências [$t(104) = 9.45$, $p < .001$], e no escore composto de desempenho acadêmico [$t(104) = 12.70$, $p < .001$]. É importante notar que 85% dos maus leitores apresentaram uma baixa pontuação em escrita (menor ou igual a 5); 72% tiveram pontuação insuficiente em matemática, 59% tiveram dificuldades em linguagem oral, 64% apresentaram dificuldades em ciências. No grupo de bons leitores, as porcentagens de crianças que apresentaram desempenho igual ou menor a 5 foram os seguintes: 9% para escrita, 4% para matemática, 2% para linguagem oral e 2% para ciências.

DESENVOLVIMENTO DOS TESTES

No Brasil, há uma escassez de testes padronizados que podem ser utilizados para avaliar as funções executivas em crianças pequenas. Os autores fizeram uma revisão crítica de um grande número de instrumentos nacionais e internacionais e discutiram os achados com um grupo de pesquisadores, psicólogos e professores experientes na área. A seleção das tarefas baseou-se em aspectos teóricos. O material foi cuidadosamente adaptado ou desenvolvido para o contexto Brasileiro, e foi feito um piloto em uma população Brasileira. As instruções das tarefas originárias de testes publicados em Inglês foram traduzidas para o Português por um membro da equipe de pesquisa (CJT), que é um Brasileiro nato fluente em Inglês. As traduções, em conjunto com seus respectivos originais em Inglês, foram revisadas por um grupo de cinco assessores independentes experientes fluentes tanto em Português quanto em Inglês, e as melhores características das diferentes revisões foram mantidas. As medidas foram testadas previamente e os itens problemáticos foram modificados pelo grupo, incluindo o tradutor original. A fidedignidade dos instrumentos foi medida e reportada na seção de resultados. Um resumo dos testes de funções executivas utilizados na pesquisa, bem como o respectivo componente executivo que se pretendia medir está listado na **Tabela 2** e são descritos em detalhes abaixo.

PROCEDIMENTO

O estudo foi aprovado pelos Comitês de Ética em Pesquisa da Universidade de Luxemburgo, Universidade Federal de São Paulo, Hospital das Clínicas da Faculdade de Medicina da Universidade de São Paulo e Maternidade Climério de Oliveira da Universidade Federal da Bahia, assim como foi obtida a aprovação da Comissão Nacional de Ética em Pesquisa (CONEP). Todos os participantes tiveram seus termos de consentimento livre e esclarecidos assinados pelos pais ou cuidadores. Cada criança foi avaliada individualmente em uma área calma da escola em duas sessões realizadas em dias diferentes, com duração de aproximadamente uma hora cada. Intervalos curtos foram inseridos no meio das sessões para manter a motivação das crianças. As medidas foram aplicadas em uma sequência fixa determinada previamente para variar a natureza das demandas requeridas pelos testes. As crianças receberam um adesivo após completar cada fase da bateria de avaliação e ganharam um diploma colorido ao final pela participação na pesquisa. Elas foram testadas por oito pesquisadores assistentes que foram treinados por um membro da equipe de pesquisa (PEdA). No total, as crianças completaram uma bateria de 19 tarefas cobrindo o funcionamento executivo e outros domínios cognitivos; os resultados dos 12 testes de funções executivas estão reportados aqui. O funcionamento executivo foi avaliado por tarefas computadorizadas ou envolvendo material impresso.

Para todas as medidas, a pontuação foi convertida em escores T usando a média amostral e desvio padrão da amostra completa de 355 crianças Brasileiras. Os sinais dos escores das variáveis cuja menor pontuação indicava melhor desempenho foram invertidos, de forma que todas as pontuações positivas representassem desempenho superior.

Tabela 2 | Medidas de funções executivas selecionadas para este estudo.

Componente Hipotético das Funções Executivas	Tarefas
Flexibilidade cognitiva	Tarefa do Pato modificada a partir do *Dimensional Change Card Sort* (Zelazo, 2006)
	Opposite Worlds do *Test of Everyday Attention for Children* (Manly et al., 1998)
Memória operacional	*Digit Recall* do *Automated Working Memory Assessment* (Alloway, 2007)
	Counting Recall do *Automated Working Memory Assessment* (Alloway, 2007)
	Dot Matrix do *Automated Working Memory Assessment* (Alloway, 2007)
	Odd-One-Out do *Automated Working Memory Assessment* (Alloway, 2007)
Inibição	O Mestre Mandou (*Simon says*)
	Go/No-Go modificado de Cragg & Nation (2008)
	Simon task
	Flanker task modificado do *Attention Network task* de Rueda et al. (2004)
Atenção seletiva	*Map Mission* do *Test of Everyday Attention for Children* (Manly et al., 1998)
	Sky Search do *Test of Everyday Attention for Children* (Manly et al., 1998)

MEDIDAS

Resolução não verbal

As crianças completaram as Matrizes Progressivas Coloridas de Raven (Raven, Court, e Raven, 1986) na qual é necessário completar uma figura geométrica a partir da escolha do pedaço que está faltando, dentre seis desenhos diferentes. Os padrões aumentam progressivamente em dificuldade e o teste consiste em 36 itens. As normas da população Brasileira estão disponíveis (Angelini, Alves, Custódio, Duarte, e Duarte, 1999).

Flexibilidade cognitiva

Duas medidas de flexibilidade cognitiva foram utilizadas: a Tarefa do Pato e a tarefa *Opposite Worlds*. Ambas as atividades continham diferentes condições e as crianças tinham que alternar entre uma condição e outra.

A Tarefa do Pato é uma versão do *dimensional change card-sorting task* que foi modificada de Zelazo (2006). As crianças tinham que classificar cartões bivalentes (vermelho/azul; pato/flor) de acordo com uma regra específica (cor ou forma). A regra de classificação mudava ao longo do teste, mas os cartões de estímulos eram os mesmos, com cada cartão representando sempre as duas dimensões simultaneamente. Dois cartões alvo (um pato azul e uma flor vermelha) foram anexados às bandejas de classificação e permaneceram visíveis durante toda a execução do teste. Os cartões tinham que ser colocados em uma das duas bandejas, com a face do desenho virada para baixo. Inicialmente, as crianças eram solicitadas a classificar os cartões de acordo com a forma (jogo da forma), e em seguida pela cor (jogo da cor). Em cada caso, o avaliador explicava a regra de classificação e demonstrava com dois exemplos. A criança então realizava duas tentativas para prática com *feedback*, seguido de seis itens sem *feedback*, que consistiam no experimento em si. Na última tarefa, cartões condicionais, que continham um adesivo de estrela adicional foram introduzidos. As crianças eram solicitadas a classificar os cartões pela cor quando havia a estrela colada, e pela forma quando não havia uma estrela colada (jogo da cor e forma). O avaliador demonstrava em dois exemplos (um com estrela) e verificava verbalmente se a criança havia compreendido as regras do jogo. Novamente, as crianças realizavam duas tentativas para prática com *feedback* (uma com estrela). Se a criança falhasse na prática, o avaliador repetia as regras do jogo e a criança completava duas práticas adicionais sem *feedback*. Depois destes itens de prática, as regras eram retomadas e os itens experimentais começavam. As crianças tinham que classificar 24 cartões, sendo que havia um lembrete das regras após 12 itens, sem qualquer *feedback*. A maioria dos cartões (16) tinha que ser classificada pela forma; um terço deles (8) continha os adesivos de estrela. No “jogo da cor” e “jogo da forma” as crianças obtiveram pontuação máxima (efeito teto). O número de classificações corretas dos itens com adesivos de estrela no “jogo da cor e forma” foi usado como variável dependente nas análises.

A tarefa *Opposite Worlds* constitui parte do *Test of Everyday Attention for Children* (Manly, Robertson, Anderson, e Nimmo-Smith, 1998). Foi apresentada às crianças uma prancha de estímulos contendo uma trilha dos dígitos um e dois. Na condição “mundo igual”, elas tinham que seguir a trilha e nomear os dígitos o mais rápido possível, da forma convencional. Na condição “mundo ao contrário”, elas eram solicitadas a dizer “dois” para o dígito um e “um” para o dígito dois enquanto seguiam a trilha com os dedos. O teste começa com a condição “mundo igual”, seguida por duas condições do “mundo ao contrário”, e uma nova condição do “mundo igual”. A variável dependente usada para as análises foi a soma de respostas corretas em todas as condições.

Memória operacional

A memória operacional foi avaliada usando quatro subtestes do teste computadorizado *Automated Working Memory Assessment* (AWMA, Alloway, 2007). As medidas são de *span* verbal ou visuoespacial em que o número de itens a ser memorizado aumenta progressivamente ao longo de blocos sucessivos contendo seis tentativas cada. O teste é interrompido após três erros em um mesmo bloco e o número de itens lembrados corretamente consiste na variável dependente.

Duas medidas de memória operacional verbal foram utilizadas: *Digit Recall* e *Counting Recall*. O *Digit Recall* é uma tarefa de *span* simples em que a criança deve repetir imediatamente, e na mesma ordem apresentada, algumas sequências de dígitos. Na tarefa *Counting Recall* (uma atividade de *span* complexo), as crianças tem que contar e memorizar o número de círculos em pranchas que contém desenhos de círculos e triângulos. No final de cada bloco, o número de círculos em cada prancha tem que ser evocado na ordem correta.

As crianças também completaram duas medidas de memória operacional visuoespacial: *Dot Matrix* e *Odd-One-Out*. Na tarefa de *span* simples *Dot Matrix*, as crianças observavam uma matriz 4X4 e um ponto vermelho aparecia em diferentes localizações, em sequência. As crianças tinham que memorizar a sequência de aparecimento dos pontos vermelhos e evocá-la corretamente em seguida, apontando para os quadrados em branco da matriz. A tarefa *Odd-One-Out* um teste de *span* complexo no qual as crianças veem pequenas matrizes de três "caixas" com uma forma dentro de cada. Duas destas formas são sempre idênticas. As crianças têm que identificar a forma que não combina, memorizar sua localização em cada matriz e evocar a localização de todas as formas diferentes, na sequência correta. A evocação é feita a partir de matrizes em branco, ao final de cada bloco.

Inibição

A inibição de resposta foi avaliada por dois testes (O Mestre Mandou e *Go/No-Go*) nos quais apenas em determinadas condições a criança deveria apresentar uma resposta motora, enquanto outras deveriam ser ignoradas. As crianças também completaram o *Simon Task* e o *Flanker Task*, que avaliam a supressão de interferência. Nestas tarefas, as características dos aparatos bivalentes podiam converter para uma resposta única (itens congruentes) ou gerar conflito por sugerir diferentes respostas (itens incongruentes).

Na tarefa "O Mestre Mandou", uma versão Brasileira do jogo *Simon says*, as crianças tinham que ficar de pé em frente ao avaliador, que desempenhavam uma série de ações físicas acompanhadas por comandos verbais (e.g. "mãos na cabeça"). As crianças tinham que imitar as ações do avaliador se o comando verbal fosse precedido pela expressão "o mestre mandou", mas deviam ficar imóveis se o comando não começasse com "o mestre mandou". O avaliador desempenhava todas as ações, independentemente do comando verbal dado. No total, 16 tentativas foram realizadas, das quais oito correspondiam aos itens em que a criança não deveria imitar o avaliador. O teste foi precedido por duas tentativas de prática, com *feedback*, e as crianças foram relembradas sobre as regras ao final da primeira metade da fase experimental. A medida dependente utilizada para as análises foi a soma de respostas nos itens em que a criança não deveria imitar o avaliador, os quais podiam ter sido pontuados como 3 (nenhum movimento), 2 (movimento errado), 1 (imitação parcial), e 0 (imitação completa).

A tarefa *Go/No-Go* foi uma versão adaptada do teste Inglês usado por Cragg e Nation (2008). A tarefa é apresentada em um computador *laptop* e consiste em uma cena de fundo representando um jogo de futebol, com uma bola de futebol (itens *Go*) ou futebol americano (itens *No-Go*) que surge na marca do pênalti (região central inferior da tela) por 200ms. As crianças eram instruídas a manter apertado o botão esquerdo do mouse embutido no computador (marcado com uma estrela) com o dedo indicador de sua mão preferencial. Quando a bola de futebol aparecia na tela, a criança tinha que "chutá-la", e para isso deveria soltar o botão da estrela e apertar o botão direito do mouse o mais rápido possível, com o mesmo dedo indicador. Quando uma bola de futebol americano apareceria na tela, elas deveriam manter o indicador pressionando o botão da estrela para não "chutar" a bola esquisita. As crianças completaram inicialmente dois blocos de 10 itens *Go* cada. Em seguida, foram apresentados dois blocos misturados (contendo itens *Go* e *No-Go*) de 32 itens cada. Os estímulos *No-Go* apareciam em 25% das tentativas. A medida dependente usada para a análise foi a porcentagem de respostas corretas nos blocos misturados. Os itens *Go* foram pontuados como corretos se a criança soltasse completamente o botão da estrela e apertasse o botão adjacente à direita. Os itens *No-Go* foram pontuados como corretos se a criança continuasse pressionando o botão da estrela.

As tarefas do *Simon* e *Flanker* também foram feitas em um *laptop* e foram programadas com o software E-Prime 2.0 (Psychology Software Tools, Pittsburgh, PA). As respostas foram registradas com dois botões circulares coloridos de resposta (diâmetro de 2.5"), que foram colocados próximos aos cantos inferiores esquerdo e direito do teclado do *laptop*. No *Simon task*, ursos verdes e amarelos (2.75" X 2.56") apareciam do lado esquerdo ou direito da tela. As crianças tinham que apertar o botão verde para o urso verde, e o botão amarelo para o urso amarelo, o mais rápido possível, independentemente da

posição em que o urso aparecesse. Metade das tarefas eram incongruentes, e o urso aparecia no lado oposto ao do botão apropriado. O *Flanker task* foi uma versão adaptada do *Attention Network Task* de Rueda et al. (2004). Uma linha horizontal de cinco peixes amarelos separados pela mesma distância era apresentada (3.35" X 0.39") e as crianças tinham que indicar a direção do peixe central "Nemo" ao apertar o botão correspondente (da esquerda ou direita) o mais rápido possível. Nos itens congruentes (50% das tentativas), os peixes apontavam todos para a mesma direção do peixe-alvo, e nos itens incongruentes (50% das tentativas), os distratores apontavam para a direção oposta.

Em ambas as tarefas (Simon e Flanker), as tentativas iniciavam com uma cruz de fixação no meio da tela por um segundo, seguida pelo estímulo por cinco segundos ou até que a resposta fosse dada. As respostas eram seguidas por um *feedback* e um intervalo vazio de 400ms. Dois blocos de 20 itens cada tinham que ser completados, e a apresentação dos itens congruentes e incongruentes era randomizada. Oito itens de prática precediam os itens experimentais. Se mais do que dois erros ocorressem nestas tentativas, as instruções e a prática eram repetidas até a criança atingir o nível esperado. As medidas dependentes utilizadas para as análises foram os tempos de reação (TR) nos itens incongruentes (excluindo respostas incorretas, TR abaixo de 200 ms e TE abaixo de 3 DP da média do próprio sujeito).

Atenção seletiva

Duas tarefas de busca visual cronometrada do *Test of Everyday Attention for Children* (Manly, Robertson, Anderson, e Nimmo-Smith, 1998) foram utilizadas: *Map Mission* e *Sky Search*.

Na tarefa *Map Mission*, as crianças recebiam um mapa de uma cidade em folha A3 contendo vários distratores (e.g. pequenos símbolos de carrinhos de supermercado, carros, etc). Elas tinham que circular o maior número possível de desenhos-alvo (pequenos símbolos de bombas de gasolina) em um minuto, com uma caneta de marcação. No total, havia 80 desenhos-alvo na folha. A variável dependente era o número de desenhos-alvo circulado.

Na tarefa *Sky Search*, as crianças recebiam uma folha A3 com 128 espaçonaves pareadas, das quais 20 pares eram idênticos. Elas tinham que circular apenas os pares idênticos, o mais rápido possível, com uma caneta de marcação. Em seguida, as crianças completavam uma versão de controle motor da mesma tarefa, contendo apenas os 20 estímulos-alvo. Para ambas as versões do teste, as crianças tinham que marcar um X no quadrado no canto da página quando tivessem acabado, e a marcação do tempo era interrompida. A pontuação do controle motor (tempo sobre alvo) era subtraída da pontuação inicial no teste, gerando um escore final no *Sky Search* que é relativamente independente de variações na velocidade motora.

Avaliações dos professores em sala de aula

Os professores foram solicitados a avaliar o desempenho acadêmico das crianças ao longo do ano em uma escala de 1 a 10 nas seguintes áreas: leitura: decodificação e compreensão; escrita: ortografia, redação e caligrafia; matemática: numeração, contas aritméticas e compreensão de problemas; linguagem oral: expressão e compreensão; ciências humanas e da natureza: ciências, história e geografia. Os escores compostos foram calculados para escrita, matemática, linguagem oral e ciências humanas e da natureza, a partir da extração da média dos diferentes subescores em cada domínio. Para cada aluno, o desempenho total também foi calculado.

Tabela 3 | Estatística descritiva para os escores de raciocínio não verbal e funções executivas (N = 106).

Medidas	Média	*DP*	Faixa	α Cronbach
**Raciocínio não verbal (percentil)**				
MPC Raven	53.37	27.98	10-99	0.82
**Flexibilidade cognitiva**				
Tarefa do Pato	46.03	9.52	34.19-63.42	0.87
Opposite Worlds	44.93	9.79	20.06-62.74	0.66
**Memória operacional**				
Digit recall	45.42	9.84	22.01-76.81	0.93
Counting recall	45.48	9.58	30.00-72.89	0.92
Dot matrix	46.58	9.55	23.08-68.95	0.91
Odd-one-out	46.47	9.88	30.88-75.74	0.91
**Inibição**				
Mestre mandou	49.42	11.14	20.86-65.77	0.6
Go/No-Go	50.11	10.38	26.92-69.34	0.73
Simon task	46.94	9.09	24.82-73.22	0.83
Flanker task	47.53	10.21	27.38-74.22	0.87
**Atenção seletiva**				
Map Mission	48.29	8.43	30.19-78.78	N/A
Sky Search	47.55	9.69	30.99-75.95	N/A

MPC Raven: Matrizes Progressivas Coloridas de Raven. Com exe- -ção do Raven, todos os escores são T-escores. α Cronbach não foi computada para as medidas de tempo das tarefas de atenção seletiva.

RESULTADOS

ESTATÍSTICAS DESCRITIVAS E ANALISES DE CORRELAÇÃO

Não houve perda de dados e nenhuma das variáveis manifestou desvios significativos da normalidade (Kline,

2005). Estatística descritiva para as medidas de raciocínio não verbal e funções executivas estão apresentadas na **Tabela 3**. A confiabilidade interna calculada para a pontuação nas diferentes medidas foi estabelecida com base na amostra total (N=355), utilizando o Alpha de Cronbach. Os coeficientes de confiança ficaram dentro de uma variabilidade média aceitável entre os níveis entre .60 e .93.

Os coeficientes de correlação simples (ordem-zero) entre idade, raciocínio não verbal e as diferentes medidas de funções executivas, estão descritas no triângulo superior da **Tabela 4**. O triângulo inferior demonstra as correlações parciais controladas pela idade cronológica (em meses). O padrão global das relações não modificou quando a idade foi controlada parcialmente. Como existe uma grande sobreposição entre inteligência fluída e funcionamento executivo (Kyllonen e Christal, 1990; Engle, Tuholski, et al., 1999; Conway, Cowan, Bunting, Therriault, e Minkoff, 2002; Colom, Flores-Mendoza, e Rebollo, 2003; Kane et al., 2004; Engel de Abreu, Conway, e Gathercole, 2010), a medida de raciocínio não verbal não foi usada como covariável quando explorada a relação entre os componentes das funções executivas (Dennis et al., 2009).

Como esperado, raciocínio não verbal foi significativamente relacionado com todas as medidas de funções executivas com exceção da tarefa Go/no Go (*r* variando de .24 a .43). Entre os domínios de memória operacional e atenção seletiva, as medidas apresentaram correlação significativa (*r* variando entre .38 e .53), e as correlações que demonstram validade convergente foram mais elevadas que as correlações que demostraram validade discriminante. Também foi observada uma correlação significativa entre as duas medidas de flexibilidade cognitiva (*r* =.23). Estas tarefas também tiveram correlação moderada com outros domínios das funções executivas, especialmente com memória operacional (*r* variando entre .26 e .37). Para inibição, foi observada alta correlação entre as tarefas de supressão de interferência, Tarefa do Simon e Tarefa do Flanker (*r* = .64), e uma correlação significativa entre as medidas de resposta inibitória Mestre mandou e Go/no Go (*r* = .25). Particularmente, intercorrelações entre as medidas de supressão de interferência e resposta inibitória foram baixa (*r* variando de .07 a .20). Dentre os construtos das funções executivas, as medidas de memória operacional manifestaram a maior correlação, enquanto os demais

Tabela 4 │Correlação entre idade, raciocínio não verbal e funções executivas, utilizando o coeficiente de correlação Pearson (N = 106).

Medidas	1	2	3	4	5	6	7	8	9	10	11	12	13	14
1. Idade	--	--	--	--	--	--	--	--	--	--	--	--	--	--
**Raciocínio não verbal**														
2. MPC Raven	-0.13	--	**0.35**	**0.34**	**0.43**	**0.44**	**0.47**	**0.45**	**0.26**	0.1	**0.4**	**0.36**	**0.35**	**0.26**
**Flexibilidade cognitiva**														
3. Tarefa do pato	-0.05	**0.35**	--	**0.23**	**0.26**	**0.35**	**0.26**	**0.3**	0.14	-0.07	0.13	0.04	0.02	0.09
4. Mundo ao contrário	0.04	**0.33**	**0.23**	--	**0.25**	**0.29**	**0.36**	**0.38**	**0.25**	**0.19**	**0.28**	0.17	**0.23**	**0.28**
**Memória operacional**														
5. Digit recall	0.1	**0.41**	**0.26**	**0.26**	--	**0.52**	**0.39**	**0.38**	**0.31**	0.14	**0.23**	0.18	**0.25**	**0.22**
6. Counting recall	0.17	**0.41**	**0.34**	**0.29**	**0.53**	--	**0.38**	**0.49**	**0.25**	0.03	**0.28**	**0.25**	**0.23**	**0.33**
7. Dot matrix	**0.21**	**0.43**	**0.24**	**0.35**	**0.39**	**0.4**	--	**0.5**	**0.19**	0.18	**0.39**	**0.3**	**0.2**	0.1
8. Odd-one-out	**0.31**	**0.38**	**0.27**	**0.37**	**0.38**	**0.52**	**0.53**	--	**0.26**	0.03	**0.43**	**0.45**	0.11	0.2
**Inibição**														
9. Mestre mandou	0.1	**0.24**	0.14	**0.25**	**0.31**	**0.26**	**0.21**	**0.28**	--	**0.23**	0.17	**0.2**	**0.17**	0.15
10. Go/No-Go	0.17	0.1	-0.07	**0.2**	0.15	0.06	**0.21**	0.08	**0.25**	--	0.05	0.06	0.15	0.11
11. Tarefa do Simon	0.17	**0.37**	0.12	**0.28**	**0.24**	**0.3**	**0.41**	**0.45**	0.18	0.07	--	**0.64**	0.17	0.22
12. Tarefa do Flanker	0.1	**0.35**	0.03	0.17	0.18	**0.26**	**0.3**	**0.45**	**0.2**	0.07	**0.64**	--	**0.25**	0.19
**Atenção seletiva**														
13. Missão do mapa	**0.32**	**0.29**	0	**0.23**	**0.26**	**0.27**	**0.25**	**0.2**	0.19	**0.2**	**0.22**	**0.26**	--	**0.36**
14. Procura no céu	0.18	**0.24**	0.08	**0.28**	**0.23**	**0.35**	0.13	**0.24**	0.16	0.14	**0.24**	0.17	**0.39**	--

MPC Raven: Matrizes Progressivas Coloridas de Raven. Triângulo superior demonstra correlações de primeira ordem, e o triângulo inferior demonstra correlações controlando por idade em meses. p<.05 estão marcadas em negrito.

domínios das funções executivas e medidas de resposta inibitória apresentaram as correlações mais baixas.

COMPONENTES DE FUNCIONAMENTO EXECUTIVO

As 12 tarefas de funções executivas foram submetidas a análise de componentes principais com rotação Varimax da estrutura fatorial. Quatro fatores com *autovalores* (i.e., *eigenvalues*) acima de 1.00 foram extraídos e representaram 62.50% da variância total. As cargas fatoriais com rotação Varimax estão apresentadas na **Tabela 5**. Carga acima de .40 foi utilizada como critério para interpretação dos fatores. As medidas de memória operacional e flexibilidade cognitiva tiveram alta carga no Fator 1 (32.7%, carga fatorial entre .41 e .78). As medidas de supressão de interferência (Simon e Flanker) tiveram alta carga no Fator 2 (10.8%, carga fatorial de .85 e .87), e também carga moderada nas tarefas de memória operacional visuoespacial (carga fatorial entre .46 e .57). No Fator 3 (10.5%) foram incluídos os subtestes de atenção seletiva (carga fatorial de .75 e .83). As medidas de resposta inibitória tiveram alta carga no Fator 4 (8.5%, carga fatorial de .61 e .85). Apenas as medidas de memória operacional visuoespacial tiveram cargas acima de .40 em mais de um fator.

Tabela 5 | Carga fatorial da análise de componentes principais.

Medidas	Fator 1	Fator 2	Fator 3	Fator 4
	"Memória operacional/ Flexibilidade cognitiva"	*"Supressão de interferência"*	*"Atenção seletiva"*	*"Resposta inibitória"*
Tarefa do pato	**0.78**	-0.04	-0.11	-0.10
Mundo ao contrário	**0.41**	0.19	0.21	0.37
Digit recall	**0.62**	0.11	0.24	0.28
Counting recall	**0.70**	0.22	0.35	0.05
Dot matrix	**0.47**	**0.46**	0.00	0.35
Odd-one-out	**0.54**	**0.57**	0.10	0.15
Mestre mandou	0.27	0.11	0.06	**0.61**
Go/No-Go	-0.12	0.00	0.12	**0.85**
Tarefa do Simon	0.13	**0.85**	0.14	0.05
Tarefa do Flanker	0.00	**0.87**	0.14	0.05
Missão do mapa	0.03	0.18	**0.75**	0.21
Procura no céu	0.18	0.07	**0.83**	0.02

Carga fatorial acima de .40 estão marcadas em negrito.

Os quatro componentes extraídos foram nomeados como "Memória operacional/ Flexibilidade cognitiva" (Fator 1), "Supressão de interferência" (Fator 2), "Atenção seletiva" (Fator 3) e "Resposta inibitória" (Fator 4). Método de regressão foi utilizado para os escores produzidos para cada fator participante nesta solução, e foram utilizados como medidas dependentes nas análises subsequentes.

RELAÇÃO ENTRE OS COMPONENTES DO FUNCIONAMENTO EXECUTIVO E AVALIAÇÃO DOS PROFESSORES

A **Tabela 6** apresenta os coeficientes das correlações parciais entre a estrutura dos fatores das funções executivas identificados e a avaliação dos professores, controlados pela idade cronológica. A avaliação do desempenho acadêmico teve alta correlação entre as áreas avaliadas (*r* variando de .80 a .98). A correlação entre decodificação e compreensão de leitura foi a maior (*r* = .98).

O Fator 1 teve correlação moderada a alta com todas as áreas da classificação de desempenho do professor (*r* variando de .29 a .43). O Fator 2 teve correlação significativa com leitura, escrita e matemática (*r* variando de .20 a .29) e o Fator 3 foi significativamente relacionado com a área de leitura (*r* variando de .22 a .25) e linguagem oral (*r* de .25). Baixa correlação foi identificativa entre Fator 4 e a classificação nas áreas de decodificação e escrita (*r* de .22).

Considerando a classificação do desempenho em leitura, a correlação mais forte foi observada com Fator 1 (*r* variando entre .35 e .36). Estas correlações foram particularmente maiores do que as relações de leitura com os demais fatores das funções executivas (*r* variando de .18 a .25) e se mantiveram significativas mesmo após controle por raciocínio não verbal (*r* variando de .21 a .34).

DESEMPENHO DE LEITORES COM DIFICULDADES E LEITORES EFICIENTES NOS COMPONENTES DE FUNCIONAMENTO EXECUTIVO

Uma série de Análises de Covariância foram realizadas com a pontuação do fator de funções executivas utilizada como variável dependente. Após ser realizado o controle por idade cronológica, diferenças significativas entre grupos foram observadas no fator Memória operacional/ Flexibilidade cognitiva [$F(1, 103) = 9.29$; $p < .01$], com o grupo de leitores eficientes superando o desempenho do grupo de leitores com dificuldade [leitores com dificuldade: $M = -.28$, $DP = 1.01$; leitores eficientes: $M = .28$, $DP = .91$]. Este efeito de grupo se manteve significativo mesmo após controlar por raciocínio não verbal [$F(1, 102) = 4.05$; $p < .05$]. O desempenho entre grupos foi equivalente nos demais fatores.

Análise de regressão logística foi conduzida para predizer a probabilidade de pertencer a um dos dois grupos de leitura utilizando Memória operacional/ Flexibilidade cognitiva como preditor. Um teste de modelo completo versus o modelo apenas com intercepto foi estatisticamente significativo, indicando que a confiabilidade do Fator de Memória operacional/ Flexibilidade cognitiva difere entre os leitores com dificuldades e leitores eficientes [$\chi^2(1) = 8.64$; $p < .01$). O poder de predição no geral foi de 61.3%:

Tabela 6 | Correlações parciais (idade em meses como covariável) entre a estrutura dos fatores das funções executivas identificados e a avaliação dos professores, utilizando coeficientes de correlação de Pearson (N = 106).

Classificação Professores	Fator 1	Fator 2	Fator 3	Fator 4	1	2	3	4	5	6	7
1. Decodificação	**0.35***	**0.20**	**0.22**	**0.22**	--						
2. Compreensão de leitura	**0.36***	**0.20**	**0.25***	0.18	**0.98**	--					
3. Escrita	**0.29***	**0.25**	0.18	**0.22**	**0.86**	**0.90**	--				
4. Matemática	**0.43***	**0.29***	0.18	0.14	**0.88**	**0.87**	**0.86**	--			
5. Linguagem Oral	**0.42***	0.18	**0.25**	0.13	**0.83**	**0.85**	**0.80**	**0.85**	--		
6. Ciências	**0.40***	0.19	0.19	0.17	**0.80**	**0.82**	**0.82**	**0.87**	**0.88**	--	
7. Escore composto	**0.41***	**0.23**	**0.22**	0.18	**0.91**	**0.92**	**0.91**	**0.94**	**0.93**	**0.96**	--

*Fator 1 "Memória operacional/ Flexibilidade cognitiva", Fator 2 "Supressão de interferência", Fator 3 "Atenção seletiva" e Fator 4 "Resposta inibitória". p < .05 estão marcados em negrito. *coeficientes de correlação que permaneceram significativos após controlar por raciocínio não verbal.*

66% foi classificado corretamente para o grupo de leitores com dificuldades e 57% para o grupo de leitores eficientes. O critério de Wald demonstrou que o Fator Memória operacional/ Flexibilidade cognitiva teve uma contribuição significativa como preditor (p <.01).

DISCUSSÃO

Este estudo investigou o funcionamento executivo e desempenho em leitura de crianças Brasileiras entre 6 e 8 anos. Aspectos positivos desta pesquisa incluem a heterogeneidade da amostra (previamente selecionada de uma grande diversidade socioeconômica e de desempenho em leitura), a utilização de diversas tarefas que medem em diferentes componentes das funções executivas, e o cuidadoso pareamento dos grupos de participantes em importantes fatores sociodemográficos. Inicialmente os achados mostram que para esta população de crianças, diferenças individuais nos componentes de funcionamento executivo contribuem de maneira distinta para o desempenho precoce em leitura. Além disso, naquelas crianças que a avaliação da performance em leitura foi classificada pelos professores como abaixo da média e apresentam dificuldade em memória operacional/ flexibilidade cognitiva quando comparados aos leitores eficientes.

A distinção entre diferentes componentes das funções executivas corrobora os estudos previamente realizados com adultos (Robbins, 1996; Miyake et al., 2000; Friedman et al., 2008) e crianças (Lehto et al., 2003; Senn et al., 2004; Huizinga et al., 2006; St Clair-Thompson e Gathercole, 2006; Van der Sluis et al., 2007), e é consistente com a teoria de multicompentes do funcionamento executivo (Miyake et al., 2000). Neste estudo com crianças brasileiras os seguintes componentes de funções executivas foram identificados: (1) Memória operacional/ Flexibilidade cognitiva, (2) Supressão de interferência, (3) Atenção seletiva, (4) e Resposta inibitória. Notavelmente, as medidas de flexibilidade cognitiva não levaram à distinção de um construto subjacente das funções executivas. Ao contrário, compartilharam uma associação em comum com as medidas de memória operacional. Estes achados se diferenciam dos estudos realizados com adultos (Miyake et al., 2000), mas estão em consonância com outros estudos com crianças, indicando que a flexibilidade cognitiva pode ser menos diferenciável da memória operacional em crianças pequenas quando comparada a diferenciação observada em estudos com crianças maiores e adultos (Senn et al., 2004; St Clair-Thompson e Gathercole, 2006). É importante destacar que a flexibilidade cognitiva pode sim existir como um construto latente, mas pode ser difícil identificar numa análise fatorial exploratória porque este construto pode não responder por uma parcela significativa da variância que não seja compartilhada com as medidas de memória operacional.

Um outro dado não esperado foi que as tarefas de supressão de interferência e resposta inibitória não foram relacionadas, indicando que estas medidas capturam diferentes aspectos do controle inibitório. Este achado amplia dados já previamente descritos em Martin-Rhee and Bialystok (2008), e é consiste com a visão de que existe diferentes componentes inibitórios (Barkley, 1997; Nigg, 2000; Friedman e Miyake, 2004). Os resultados demonstraram que as tarefas de memória operacional verbal e visuoespacial, como também as tarefas de span simples (i.e., memória de curto prazo) e tarefas de memória operacional de span complexo, relacionaram-se com o mesmo fator subjacente, demonstrando que estas medidas dependem, pelo menos em parte, de recursos executivos de domínio geral em crianças mais novas. Tarefas de memória operacional visuoespacial foram também relacionadas com as medidas de supressão de interferência. Estes achados corroboram modelos teóricos em adultos que a habilidade em lidar com interferência ou conflito representa um

componente essencial da capacidade de memória operacional (Oberauer e Kliegl, 2001; Braver, Gray, Burgess, 2007; Hasher, Lustig, e Zacks, 2007; Kane, Conway, Hambrick e Engle, 2007; Unsworth e Engle, 2007).

Estes resultados são consistentes com pesquisas previamente realizadas em países de língua inglesa, que relatam sobre a contribuição independente de componentes distintos das funções executivas para o desempenho acadêmico das crianças (St Clair-Thompson e Gathercole, 2006), e também se aplica aos achados em população de crianças brasileiras. O fator Memória operacional/ Flexibilidade cognitiva foi o melhor preditor de desempenho em leitura e a magnitude desta relação foi consideravelmente maior que as associações encontradas entre leitura e outros componentes executivos. Foi observado que a Memória operacional/ Flexibilidade cognitiva permaneceu bastante associada aos escores de leitura mesmo quando controlado pelo raciocínio não verbal. Estes resultados validam a contribuição que a capacidade de memória operacional fornece como base de sustentação para o desenvolvimento de habilidades prévias relacionadas à alfabetização (Swanson e Sachse-Lee, 2001; Gathercole et al., 2006a,b; St Clair-Thompson e Gathercole, 2006; Welsh et al., 2010; Swanson et al., 2011) e demonstra que esta relação se mantém para leitores iniciantes do português do Brasil.

Memória operacional/ Flexibilidade cognitiva também foi bastante relacionada ao desempenho em outros domínios acadêmicos, especialmente em matemática. Este resultado é consistente com a visão de que a memória operacional é um gargalo para a aprendizagem, e dá suporte ao progresso acadêmico no geral, mais do que apenas na aquisição de habilidades e conhecimentos em domínios específicos (St Clair-Thompson e Gathercole, 2006). Segundo Swanson e colaboradores (Swanson e Saez, 2003; Swanson e Beebe-Frankenberger, 2004), memória operacional e desempenho escolar estão relacionados porque quanto maior os recursos em memória operacional, mais fácil é a manutenção ativa da informação e a integração desta com *inputs* (e.g., entradas) recentes e conhecimento já adquirido, que representam processo essencial para a aprendizagem acadêmica. Muitas salas de aula são ambientes com alta demanda ao sistema de memória operacional porque frequentemente as crianças devem armazenar informações em mente enquanto estão engajadas em atividades que exigem alto esforço cognitivo. O uso de instruções verbais complexas e muito extensas na sala de aula ou tarefas muito difíceis, podem levar a uma sobrecarga de memória operacional em crianças com déficits nesta habilidade. Tal situação pode resultar em falha ao completar a tarefa ou abandono da mesma - em outras palavras, uma oportunidade de aprendizagem que foi perdida – o que afeta negativamente os índices de aprendizagem (Gathercole et al., 2006b; Gathercole e Alloway, 2008).

Nosso estudo contribui às evidências já existentes que crianças com dificuldade em leitura frequentemente apresentam dificuldade em componentes específicos do funcionamento executivo. Comparados aos leitores eficientes, crianças do grupo dificuldade em leitura apresentaram escores significativamente menores no fator Memória operacional/ Flexibilidade cognitiva. Diferentemente de outros autores, nós não encontramos diferenças significativas entre os grupos em outros componentes das funções executivas (Reiter et al., 2005; Borella et al., 2010; Pimperton e Nation, 2010). Esses achados diferentes podem ser explicados pelo fato de que a maioria dos estudos prévios teve enfoque na população clínica com transtornos de aprendizagem, como a dislexia ou dificuldades específicas em compreensão. O presente estudo contou com uma amostra de crianças sem diagnóstico de transtornos de aprendizagem, e que foram classificadas como leitores com baixo desempenho em leitura pelos próprios professores.

É importante ressaltar que o enfoque deste estudo foi explorar o perfil de funcionamento executivo de crianças cujo desempenho em leitura foi classificado como abaixo da média pelos professores, e consequentemente estavam em risco de repetir o ano escolar. Uma das limitações do estudo é o potencial viés da avaliação realizada pelos professores. Seria importante se estudos futuros incluíssem testes padronizados de desenvolvimento de leitura num design de pesquisa longitudinal com objetivo de alcançar melhor compreensão da natureza da relação entre funcionamento executivo e leitura.

Este estudo teórico tem implicações práticas na área de políticas públicas. Aprender a ler vai além de uma habilidade educacional. Dificuldades em habilidades importantes para a alfabetização, somadas a um ambiente de pobreza, cria um ciclo que se auto reforça e que é difícil ser interrompido. A identificação precoce de crianças com dificuldade em leitura em conjunto com programas de remediação, que tenham foco em reduzir essas diferenças são fundamentais para contrapor-se ao impacto da pobreza na vida das pessoas. Nosso estudo sugere que muitos estudantes no Brasil podem apresentar dificuldade em leitura e apresentar baixo desempenho acadêmico devido a déficits em memória operacional. Portanto, os professores podem ter interesse em saber se os alunos com baixo rendimento acadêmico apresentam déficit em memória operacional. Ambientes de aprendizagem que evitem a sobrecarga de recursos em memória operacional podem ser um passo importante para diminuir as dificuldades em leitura observadas precocemente, e consequentemente o fracasso escolar. Pesquisas no Reino Unido têm

identificado uma variedade de métodos sobre como efetivamente administrar a demanda cognitiva na sala de aula (Gathercole et al. 2006b; Gathercole e Alloway, 2008;). Ainda deve ser investigado se tais métodos podem melhorar o potencial de aprendizagem em outros contextos culturais e educacionais. Pesquisas recentes também têm dado ênfase ao suporte direto do desenvolvimento de memória operacional com base em programas de treinamento (ver Diamond e Lee, 2011, para revisão). Uma ampla variedade de atividades tem demonstrado melhora efetiva da habilidade de memória operacional em crianças e podem auxiliar aquelas que apresentam progresso acadêmico mais lento a superar algumas dificuldades de aprendizagem (Holmes, Gathercole, e Dunning, 2009; Loosli, Buschkuehl, Perrig, e Jaeggi, 2012; Alloway, Bibile, e Lau, 2013).

Em conclusão, nosso estudo sugere que componentes específicos das funções executivas são preditores de diferenças individuais no desenvolvimento de leitura em crianças de 6 a 8 anos. Nossos dados também corroboram o conceito de que déficits em memória operacional/ flexibilidade cognitiva podem representar um fator que irá contribuir com dificuldades no desenvolvimento da leitura em crianças brasileiras.

AGRADECIMENTOS

Esta pesquisa teve o financiamento das seguintes agências de fomento: *National Research Fund* (FNR) *Luxembourg* (Grant # CO09/LM/07, Engel de Abreu e Martin); Conselho Nacional de Desenvolvimento Científico e Tecnológico (CNPq), Brasil (400857/2010-3); Fundação de Amparo à Pesquisa do Estado de São Paulo (FAPESP), Brazil (2010/11626-0 e 2010/09185-5); Coordenação de Aperfeiçoamento de Pessoal de Nível Superior (CAPES), Brasil; e Associação Fundo de Incentivo à Pesquisa (AFIP), Brasil.

Gostaríamos de agradecer todos as crianças, pais, professores e diretores das escolas participantes, pois sem eles esta pesquisa não teria sido concretizada. Salvador: Colégio Antônio Vieira, Colégio Gregor Mendel, Escola Dorilândia, Escola Municipal Ana Nery, Escola Municipal Casa da Amizade, Escola Municipal Nossa Senhora das Graças, Escola Municipal Padre José de Anchieta, Escola Municipal Senhora Santana, Escola Nova Nossa Infância, Escola Professor Bernardino Moreira, Escola Tempo de Criança; São Paulo: Colégio Anglo Brasileiro, Colégio Anhembi Morumbi, Colégio Dourado, Escola Estadual Profa. Adalgisa Moreira Pires, Escola Municipal de Ensino Fundamental Chiquinha Rodrigues, Instituto de Educação Beatíssima Virgem Maria.

Também gostaríamos de agradecer Ueslei Carneiro, Felipe Guedes, Lucas Carneiro, Manuela Sá e Adriana Rossi, pela contribuição na coleta de dados, e Anabela Cruz-Santos, Lucy Cragg e Carolina Toledo Piza pelas ricas contribuições na adaptação dos testes. Ainda, gostaríamos de agradecer aos médicos Larissa de Freitas Rezende, Maurício Costa de Abreu e Maria Celeste Miranda Costa, pelas indispensáveis orientações com relação aos critérios de exclusão médicas.

COMO CITAR ESTA PUBLICAÇÃO

Por favor cite esta publicação, como:

Engel de Abreu, P. M. J., Abreu N., Nikaedo, C. C., Puglisi, M. L., Tourinho, C. J., Miranda, M. C., Befi-Lopes, D. M., Bueno, O. F. A. & Martin, R. (2014) Executive functioning and reading achievement in school: a study of Brazilian children assessed by their teachers as "Poor Readers." *Frontiers in Psychology. 5:*550. doi: 10.3389/fpsyg.2014.00550

REFERÊNCIAS

ABEP. (2010). Critério de Classificação Econômica Brasil [Online]. Disponível em http://www.abep.org/new/criterioBrasil.aspx [Acesso em 22 de Janeiro de 2013].

Abadzi, H. (2006). *Efficient Learning for the Poor: Insights form the Frontier of Cognitive Neuroscience*. Washington: The World Bank

Alloway, T. P. (2007). *Automated Working Memory Assessment*. London, England: Pearson Assessment.

Alloway, T. P., Bibile, V., e Lau, G. (2013). Computerized working memory training: Can it lead to gains in cognitive skills in students? *Computers in Human Behavior*, *29*(3), 632–638.

Alloway, T. P., Gathercole, S.E., e Pickering, S.J. (2006). Verbal and visuo-spatial short-term and working memory in children: Are they separable? *Child Development*, *77*, 1698-1716.

Angelini, A. L., Alves, I. C. B., Custódio, E. M.; Duarte, W. F. e Duarte, J. L. M. (1999). *Matrizes Progressivas Coloridas de Raven: Escala Especial*. Manual. São Paulo: CETEPP.

Baddeley, A. D. (1996). Exploring the central executive. *The Quarterly Journal of Experimental Psychology, 49A*, 5-28.

Baddeley, A. D. (2000). The episodic buffer: A new component of working memory? *Trends in Cognitive Sciences*, *4*, 417-423.

Baddeley, A. D., e Logie, R. (1999). Working memory: The multiple component model. In A. Miyake e P. Shah (Eds.), *Models of Working Memory* (pp. 28 – 61). New York: Cambridge University Press.

Barkley, R. A. (1997). *ADHD and the Nature of Self-Control*. New York: Guilford Press.

Belintane, C. (2006). Reading and literacy in Brazil: a search beyond polarization. *Educação e Pesquisa, 32,* 261-277.

Borella, E., Carretti, B. e Pelegrina, S. (2010). The specific role of inhibition in reading comprehension in good and poor comprehenders. *Journal of Learning Disabilities, 43,* 541-552.

Braver, T. S., Gray, J. R., e Burgess, G. C. (2007). Explaining the many varieties of working memory variation: Dual mechanisms of cognitive control. In A. R. A. Conway, C. Jarrold, M. J. Kane, A. Miyake, J. N. Towse (Eds.), *Variation in Working Memory*, Oxford University Press, pp. 76-106.

Breckenridge, K., Braddick, O., e Atkinson, J. (2013). The organization of attention in typical development: A new preschool attention test battery. *British Journal of Developmental Psychology, 31,* 271–288.

Bruns, B., Evans, D., e Luque, J. (2011). *Achieving World-Class Education in Brazil: The Next Agenda.* World Bank Publications.

Cain, K. (2006). Individual differences in children's memory and reading comprehension: An investigation of semantic and inhibitory deficits. *Memory, 14,* 553–569.

Carroll, J.M., Snowling, M.J., Hulme, C., e Stevenson, J. (2003). The development of phonological awareness in preschool children.*Developmental Psychology*, *39,* 913–923.

Cartwright, K. B. (2012). Insights from cognitive neuroscience: The importance of executive function for early reading development and education. *Early Education and Development,Special Issue on Neuroscience Perspectives 23,*1-13.

Casco C, Tressoldi P. E., e Dellantonio A. (1998). Visual selective attention and reading efficiency are related in children. *Cortex, 34,* 531-46.

Colom, R., Flores-Mendoza, C., e Rebollo, I. (2003). Working memory and intelligence. *Personality and Individual Differences, 34,* 33−39.

Conway, A. R. A., Cowan, N., Bunting, M. F., Therriault, D. J., e Minkoff, S. R. B. (2002). A latent variable analysis of working memory capacity, short-term memory capacity, processing speed, and general fluid intelligence. *Intelligence, 30,* 163−183.

Cragg, L., e Nation, K. (2008). Go or no-go? Developmental improvements in the efficiency of response inhibition in mid-childhood. *Developmental Science*, *6*, 810-827.

De Beni, R., e Palladino, P. (2000). Intrusion errors in working memory tasks – Are they related to reading comprehension ability? *Learning and Individual Differences*, *12*, 131–143.

De Beni, R., Palladino, P., Pazzaglia, F., e Cornoldi, C. (1998). Increases in intrusion errors and working memory deficit of poor comprehenders. *Quarterly Journal of Experimental Psychology Section A – Human Experimental Psychology, 51*, 305–320.

Dennis M, Francis D. J, Cirino P. T, Schachar R, Barnes M. A, Fletcher J. M. (2009). Why IQ is not a covariate in cognitive studies of neurodevelopmental disorders. *Journal of the International Neuropsychological Society, 15*, 331–343.

Duff, S. C., e Logie, R. H. (2001). Processing and storage in working memory span. *Quarterly Journal of Experimental Psychology: Human Experimental Psychology, 54A*, 31–48.

Engel de Abreu, P. M. J., Conway, A., e Gathercole, S. E. (2010). Working memory and fluid intelligence in young children. *Intelligence, 38,* 552-561.

Engle, R. W., Tuholski, S. W., Laughlin, J. E., e Conway, A. R. A. (1999). Working memory, short-term memory, and general fluid intelligence: A latent-variable approach. *Journal of Experimental Psychology-General, 128*(3), 309-331.

Ehri, L. C., Nunes, S. R., Willows, D. M., Schuster, B. V., Yaghoub-Zadeh, Z., Shanahan, T., (2001). Phonemic Awareness Instruction Helps Children Learn to Read: Evidence From the National Reading Panel's Meta-Analysis. *Reading Research Quarterly, 36,* 250-287.

Fricke, S., Bowyer-Crane, C. A., Haley, A. J., Hulme, C. e Snowling, M. (2013). Efficacy of language intervention in the early years. *Journal of Child Psychology e Psychiatry, 54,* 280-290

Friedman, N. P., e Miyake, A. (2000). Differential roles for visuospatial and verbal working memory in situation model construction. *Journal of Experimental Psychology: General, 129*, 61 – 83.

Friedman, N. P., e Miyake, A. (2004). The relations among inhibition and interference control functions: A latent-variable analysis. *Journal of Experimental Psychology*, *133*, 101–135.

Ganzeboom, H. B. G. (2010, May). *A new International Socio-Economic Index (ISEI) of occupational status for the International Standard Classification of Occupation 2008 (ISCO-08) constructed with data from the ISSP 2002-2007.* Paper presented at the Annual Conference of International Social Survey Programme, Lisbon, Portugal.

Gathercole, S. E. e Alloway, T. P. (2008). *Working Memory and Learning: A Practical Guide*. London: Sage Press.

Gathercole, S.E., Alloway, T.P., Willis, C.S., e Adams, A.M. (2006). Working memory in children with reading disabilities. *Journal of Experimental Child Psychology, 93*, 265-281.

Gathercole, S.E., Lamont, E., e Alloway, T.P. (2006). Working memory in the classroom. In S. Pickering (Ed.). *Working Memory and Education,* pp. 219-240. Elsevier Press.

Hasher, L., Lustig, C., e Zacks, R. T. (2007). Inhibitory mechanisms and the control of attention. In A. Conway, C. Jarrold, M. Kane, A. Miyake, A., e J. Towse (Eds.), *Variation in Working Memory*. Pp. 227-249. New York: Oxford University Press.

Holmes, J., Gathercole, S.E., e Dunning, D.L. (2009). Adaptive training leads to sustained enhancement of poor working memory in children. *Developmental Science*, *12, F*9–F15.

Hooper, S., Swartz, C., Wakely, M., de Kruif, R., e Montgomery, J. (2002). Executive functions in elementary school children with and without problems in written expression. *Journal of Learning Disabilities, 35*, 37-68.

Huizinga, M., Dolan, C. V., e van der Molen, M. W. (2006). Agerelated change in executive function: Developmental trends and a latent variables analysis. *Neuropsychologia, 44*, 2017−2036.

Jarvis, H. L., e Gathercole, S. E. (2003).Verbal and non- verbal working memory and achievements on national curriculum tests at 11 and 14 years of age. *Educational and Child Psychology, 20*, 123-140.

Kane, M. J., Conway, A. R. A., Hambrick, D. Z., e Engle, R. W. (2007). Variation in working-memory capacity as variation in executive attention and control. In A. R. A. Conway, C. Jarrold, M. J. Kane, A. Miyake, J. Towse (Eds.), *Variation in Working Memory*. Oxford University Press.

Kane, M. J., Hambrick, D. Z., Tuholski, S. W., Wilhelm, O., Payne, T. W., e Engle, R. W. (2004). The generality of working memory capacity: A latent variable approach to verbal and visuo-spatial memory span and reasoning. *Journal of Experimental Psychology: General, 133*, 189−217.

Klenberg, L., Korkman, M., e Lahti-Nuuttila, P. (2001). Differential development of attention and executive functions in 3- to 12-year old Finnish children. *Developmental Neuropsychology, 20*, 407−428.

Kyllonen, P. C., e Christal, R. E. (1990). Reasoning ability is (little more than) working-memory capacity?! *Intelligence, 14*, 389–433.

Lan, X., Legare, C. H., Ponitz, C. C., Li, S., e Morrison, F. J. (2011). Investigating the links between the subcomponents of executive function and academic achievement: A crosscultural analysis of chinese and american preschoolers. *Journal of Experimental Child Psychology, 108,* 677-692.

Lehto, J. E., Juujärvi, Kooistra, L., e Pulkkinen, L. (2003). Dimensions of executive functioning: Evidence from children. *British Journal of Developmental Psychology, 21,* 59−80.

Locascio, G., Mahone, E.M., Eason, S.H., e Cutting, L.E. (2010). Executive Dysfunction among Children with Reading Comprehension Deficits. *Journal of Learning Disabilities, 43*, 441-54.

Loher, S. e Roebers, C. M. (2013). Executive functions and their differential contribution to sustained attention in 5- to 8-year-old children. *Journal of Educational and Developmental Psychology*, 3, 51-63.

Loosli, S.V., Buschkuehl, M., Perrig, W.J., e Jaeggi, S.M. (2012). Working memory training improves reading processes in typically developing children. *Child Neuropsychology, 18*, 62-78.

Manly, T., Anderson, V., Nimmo-Smith, I., Turner, A., Watson, P., e Robertson, I. H. (2001). The differential assessment of children's attention: The Test of Everyday Attention for Children (TEA-Ch), normative sample data and ADHD performance. *Journal of Child Psychology and Psychiatry, 42,* 1065−1081.

Manly, T., Robertson, I. H., Anderson, V., e Nimmo-Smith, I. (1998). *Test of Everyday Attention for Children*. London, England: Pearson Assessment.

Martin-Rhee, M.M., e Bialystok, E. (2008).The development of two types of inhibitory control in monolingual and bilingual children. *Bilingualism: Language and Cognition, 11,* 81-93.

Miyake, A., Friedman, N. P., Emerson, M. J., Witzki, A. H., e Howerter, A. (2000). The unity and diversity of executive functions and their contributions to complex ‘‘frontal lobe’’ tasks: A latent variable analysis. *Cognitive Psychology, 41*, 49–100.

Muter, V., C. Hulme, M. J. Snowling e J. Stevenson. (2004). Phonemes, rimes, vocabulary, and grammatical skills as foundations of early reading development: Evidence from a longitudinal study. *Developmental Psychology*, *40*, 665-681.

Nation, K., Adams, J. W., Bowyer-Crane, C. A., e Snowling, M. J. (1999). Working memory deficits in poor comprehenders reflect underlying language impairments. *Journal of Experimental Child Psychology, 73*, 139-158.

Nation, K., Clarke, P., Marshall, C. M., e Durand, M. (2004). Hidden language impairments in children: Parallels between poor reading comprehension and specific language impairment? *Journal of Speech Language and Hearing Research*, *47*, 199-211.

Nation, K., Cocksey, J., Taylor, J. S. H., e Bishop, D. V. M. (2010). A longitudinal investigation of early reading and language skills in children with poor reading comprehension. *Journal of Child Psychology and Psychiatry, 51*, 1031-1039.

National Institute of Child Health and Human Development. (2000). *Report of the National Reading Panel. Teaching children to read: An evidence-based assessment of the scientific research literature on reading and its implications for reading instruction* (NIH Publication No. 00-4769). Washington, DC: U.S. Government Printing Office.

Nigg, J. T. (2000). On inhibition/disinhibition in developmental psychopathology: Views from cognitive and personality psychology and a working inhibition taxonomy. *Psychological Bulletin*, *126*, 220–246.

Oberauer, K., e Kliegl, R. (2001). Beyond resources - Formal models of complexity effects in age differences in working memory. *European Journal of Cognitive Psychology, 13,* 187-215.

OECD. (2011). *Society at a Glance 2011 - OECD Social Indicators*, OECD publishing, (www.oecd.org/els/social/indicators/SAG)

OECD (2013). *PISA 2012 Results in Focus: What 15-Year-Olds Know and What They can do With What They Know*. OECD Publishing.

Pimperton, H., e Nation, K. (2010). Suppressing irrelevant information from working memory: Evidence for domain specific deficits in poor comprehenders. *Journal of Memory and Language, 62*, 380-391.

Pimperton, H., e Nation, K. (2012). Poor comprehenders in the classroom: teacher ratings of behavior in children with poor reading comprehension and its relationship with individual differences in working memory. *Journal of Learning Disabilities*, Aug 18. [Epub ahead of print]

Pinheiro, A. M. V. (1995). Reading and spelling development in Brazilian Portuguese. *Reading and Writing, 7,* 111–138.

PREAL. (2009). *Overcoming Inertia: A Report Card on Education in Brazil.* PREAL and the Lemann Foundation.

Rabbitt, P. (1997). Methodologies and models in the study of executive function. In P. Rabbitt

(Ed.), *Methodology of Frontal and Executive Function* (pp. 1–38). East Sussex, UK: Psychology Press Publishers.

Raven, J. C., Court, J. H., e Raven, J. (1986). *Coloured Progressive Matrices*. London, England: H. K. Lewis.

Reiter, A., Tucha, O., e Lange, K. W., (2005). Executive functions in children with dyslexia. *Dyslexia, 11,* 116–131.

Roberts, R.J. e Pennington, B.F. (1996). An interactive framework for examining prefrontal cognitive processes. *Developmental Neuropsychology, 12*, 105-126.

Romani C, Tsouknida E, di Betta AM, Olson A (2011) Reduced attentional capacity, but normal processing speed and shifting of attention in developmental dyslexia: evidence from a serial task. *Cortex, 47*, 715–733.

Rose, S. A., Feldman J. F., e Jankowski J.J., (2011). Modeling a cascade of effects: The role of speed and executive functioning in preterm/full-term differences in academic achievement. Developmental Science. 14:1161–1175.

Rueda, M. R., Fan, J., McCandliss, B. D., Halparin, J. D., Gruber, D. B., Lercari, L. P., et al. (2004). Development of attentional networks in childhood. *Neuropsychologia*, *42*, 1029-1040.

Scerif, G., Cornish, K., Wilding, J., Driver, J., e Karmiloff-Smith, A. (2004). Visual search in typically developing toddlers and toddlers with fragile X or Williams syndrome. *Developmental Science, 7*, 116-130.

Senn, T. E., Espy, K. A., e Kaufmann, P. M. (2004). Using path analysis to understand executive function organization in preschool children, 26, 445-464.

Sesma, H. W., Mahone, E. M., Levine, T., Eason S. H., e Cutting, L. E. (2009). The contribution of executive skills to reading comprehension. *Child Neuropsychology, 15*, 232–246.

Shah, P., e Miyake, A. (1996). The separability of working memory resources for spatial thinking and language processing: An individual differences approach. *Journal of Experimental Psychology: General, 125*, 4 – 27.

Sireteanu, R., Goebel, C., Goertz, R., Werner, I., Nalewajko, M., e Thiel, A. (2008). Impaired serial visual search in children with developmental dyslexia. In: Eden GF, Flower DL (eds) *Learning, skill acquisition, reading, and dyslexia*, vol 1145. Blackwell, Oxford, pp 199–211.

St Clair-Thompson, H. L., e Gathercole, S. E. (2006). Executive functions and achievements on national curriculum tests: Shifting, updating, inhibition, and working memory. *Quarterly Journal of Experimental Psychology*, 59, 745-759.

Steele, A., Karmiloff-Smith, A., Cornish, K.M. e Scerif, G. (2012). The multiple sub-functions of attention: Differential developmental gateways to literacy and numeracy. *Child Development, 83,* 2028-41.

Swanson, H. L., e Beebe-Frankenberger, M. (2004). The relationship between working memory and mathematical problem solving in children at risk and not at risk for math disabilities. *Journal of Education Psychology, 96*, 471–491.

Swanson, H. L., e Berninger, V. (1995). The role of working memory in skilled and less skilled readers' comprehension. *Intelligence, 21,* 83 – 108.

Swanson, H. L., Orosco, M. J., Lussier, C. M., Gerber, M. M., e Guzman-Orth, D. A. (2011). The influence of working memory and phonological processing on English language learner children's bilingual reading and language acquisition. *Journal of Educational Psychology, 103,* 838-856.

Swanson, H. L., e Sachse-Lee, C. (2001). A subgroup analysis of working memory in children with reading disabilities: Domain-general or domain-specific deficiency? *Journal of Learning Disabilities, 34*, 249– 263.

Swanson, H. L., e Saez, L. (2003). Memory difficulties in children and adults with learning disabilities. In H. L. Swanson, S. Graham, e K. R. Harris (Eds.), *Handbook of Learning Disabilities* (pp. 182–198). New York: Guilford.

Swanson, H. L., Saez, L., Gerber, M., e Leafstedt, J. (2004). Literacy and cognitive functioning in bilingual and nonbilingual children at or not at risk for reading disabilities. *Journal of Educational Psychology, 96,* 3 –18.

Unsworth N., e Engle, R.W. (2007). On the division of short-term and working memory: An examination of simple and complex spans and their relation to higher-order abilities. *Psychological Bulletin*, *133*, 1038-1066.

Van der Sluis, S., de Jong, P. F., e van der Leij, A. (2007). Executive functioning in children, and its relations with reasoning, reading, and arithmetic. I*ntelligence, 35*, 427-449.

Welsh, J.A., Nix, R.L., Blair, C., Bierman, K.L., e Nelson, K.E. (2010). The development of cognitive skills and gains in academic school readiness for children from low income families. *Journal of Educational Psychology, 102*, 43-53.

Wiebe, S. A., Espy, K. A., e Charak, D. (2008). Using confirmatory factor analysis to understand executive control in preschool children: I. Latent structure. *Developmental Psychology, 44,* 575–587.

World Health Organization (2007). *WHO Child Growth Standards: Methods and development: Head Circumference-for-age, Arm Circumference-for-age, Triceps Skinfold-for-age and Subscapular skinfold-for-age.* Hong Kong: WHO

Zelazo, P. D. (2006). The dimensional change card sort (DCCS): A method of assessing executive function in children. *Nature Protocols, 1*, 297-301.

Zelazo, P. D., e Carlson, S. M. (2012). Hot and cool executive function in childhood and adolescence: Development and plasticity. Child Development Perspectives, 6, 354-360.

Zelazo, P. D., Carlson, S. M., e Kesek, A. (2008). Development of executive function in childhood. In C. A. Nelson, e M. Luciana (Eds.), *Handbook of developmental cognitive neuroscience, 2nd ed.* (pp. 553-574). Cambridge, MA: MIT Press.

Conflict of Interest Statement: The authors declare that the research was conducted in the absence of any commercial or financial relationships that could be construed as a potential conflict of interest.

Received: 15 January 2014; accepted: 18 May 2014; published online: xx June 2014. Citation: Engel de Abreu PMJ, Abreu N, Nikaedo CC, Puglisi ML, Tourinho CJ, Miranda MC, Befi-Lopes DM, Bueno OFA and Martin R (2014) Executive functioning and reading achievement in school: a study of Brazilian children assessed by their teachers as "Poor Readers." Front. Psychol. **5***:550. doi: 10.3389/fpsyg.2014.00550*

This article was submitted to Developmental Psychology, a section of the journal Frontiers in Psychology.

Copyright © 2014 Engel de Abreu, Abreu, Nikaedo, Puglisi, Tourinho, Miranda, Befi-Lopes, Bueno and Martin. This is an open-access article distributed under the terms of the Creative Commons Attribution License (CC BY). The use, distribution or reproduction in other forums is permitted, provided the original author(s) or licensor are credited and that the original publication in this journal is cited, in accordance with accepted academic practice. No use, distribution or reproduction is permitted which does not comply with these terms.
